# Electrical Stimulus Controlled Binding/Unbinding of Human Thrombin-Aptamer Complex

**DOI:** 10.1038/srep37449

**Published:** 2016-11-22

**Authors:** Agnivo Gosai, Xiao Ma, Ganesh Balasubramanian, Pranav Shrotriya

**Affiliations:** 1 Department of Mechanical Engineering and Iowa State University, Ames, IA 50011, USA; 2 Microelectronics Research Center, Iowa State University, Ames, IA 50011, USA

## Abstract

The binding/unbinding of the human thrombin and its 15-mer single stranded DNA aptamer, under the application of external stimulus in the form of electrostatic potential/electric field, is investigated by a combination of continuum analysis and atomistic molecular dynamics simulation. In agreement with the experiments that demonstrate the influence of electrostatic potential on the thrombin/aptamer complex, our computations show that the application of positive electric field successfully unbinds the thrombin from the aptamer. Results from umbrella sampling simulations reveal that there is a decrease in the free energy of binding between the thrombin and aptamer in presence of positive electric fields. Hydrogen bonding and non-bonded interaction energies, and hence the free energy of binding, between the thrombin and its aptamer reduce as the applied electric field is shifted from negative to positive values. Our analyses demonstrate that application of electrical stimulus modifies the molecular interactions within the complex and consequently, electrical field can be used to modulate the association between the thrombin and its aptamer.

Structural and functional biotic-abiotic nanoscale interfaces are very promising for applications in highly sensitive, biocompatible and flexible bio-sensors and actuators. Their potential use in biomedical and biomechanical sciences include, for instance, modulation of biological systems and biomolecular activity, disease detection as well as reprogramming of genetic information^1^. However, the on-demand actuation of the system components through efficient stimulation of the biotic-abiotic interface is a challenge. Amongst the different mechanisms employed to transmit stimulus, some of the widely used methods include variable electrolyte strength^2^, temperature control^3^ and electron transfer as a result of redox reactions^4^. The practice of actuating biomolecules by electric field has been gaining interest primarily inspired by earlier findings of the influence of electrostatic field on the self-assembly and hybridization of DNA^5^,^6^,^7^, and the conformational switching of short DNA oligomers immobilized at low grafting densities^8^,^9^.

DNA aptamers used in biomedical applications, such as drug design, due to their highly specific biological activities, can potentially replace certain antibodies. They are particularly advantageous for sensors and biomedical device components because they (1) are small in size, (2) demonstrate high specificity for their receptors and (3) can be utilized for target species detection and selection of various biomolecules especially for biomedical material and drug design^10^,^11^. Thrombin is a catalytic enzyme that plays a key role during blood coagulation by synthesizing fibrin from fibrinogen and has a wide range of health effects in the human body^12^. The thrombin binding aptamer (TBA), one of the most studied synthetic oligonucleotides, shown in Fig. 1(a), is a single strand (ss) DNA with the sequence GGTTGGTGTGGTTGG^13^,^14^. TBA shows the G-quadruplex structure consisting of two planar G-quartets connected by two intervening TT and one TGT loop^15^,^16^,^17^,^18^. The G-quadruplex is stabilized through cation binding in its central binding site located between the two quadruplex planes. In addition to the active site, thrombin is characterized by two binding sites, exosite I and exosite II. The exosite I is known as the fibrinogen recognition site and the exosite II as the heparin binding site. These exosites are positively charged moieties in physiological pH and thus have the propensity of binding to negatively charged ligands, like the aptamer^11^,^13^. The two lateral TT loops of TBA bind with exosite I whereas the TGT loop has been associated with exosite II^19^. The enzymatic activity of thrombin is inhibited when bound to TBA, due to fibrinogen blocking, facilitating its potential role as an anticoagulant.

The functions of a protein depend directly on its structure, which is susceptible to applied external stimuli such as variations in: electromagnetic field, microwave radiation, temperature, pressure and pH. The observation of protein configurational change, as verified experimentally, through simulations is a challenging task, as such processes span over longer time scales of minutes whereas classical molecular dynamics (MD) simulations are in the order of much shorter timescales (~10^−9^ s or nanoseconds or ns). Nevertheless, atomistic simulations provide a strategy to deterministically explore such molecular phenomena at a very high resolution^20^,^21^. MD simulations have been previously employed to investigate the fundamental structural conformations of TBA in presence of cations (K^+^, Na^+^)^17^. Kim *et al*. carried out all atom replica exchange molecular dynamics (REMD) simulation to generate the free energy map and the folding mode of TBA^22^. Extremely long MD simulations of the order of a few microseconds concluded that the TGT loop of TBA stabilizes the entire structure of the complex in presence of the K^+^ ion and the TT loops are directly involved in the binding with the thrombin^17^. Also, Kim *et al*. computed the potential of mean force (PMF) upon pulling the aptamer molecule from the binding mode (exosite I) of the TBA/thrombin complex^22^.

We have previously reported the unbinding of TBA from thrombin in presence of an applied positive electric field through atomic force microscope (AFM) experiments^23^. The experimental setup consisted of a gold electrode which was functionalized by the thrombin aptamers (Fig. 1(b)). The TBA was attached to the gold surface with a linker and was subsequently treated with thrombin to form the complex. On application of positive electrode potential the thrombin dissociates from the aptamer layer, while in presence of low negative electric fields the complex remains in its bound state^23^. In this paper, we investigate the effects of externally applied electrostatic potential on a film of TBA in salt solution using the Poisson Boltzmann equation (PBE) and calculate the free energy of binding between TBA and thrombin by MD simulations in presence of an applied electric field. The PBE equation is utilized to characterize the electric field in the nucleic acid layer, upon the application of electrode potential, whereas the MD simulations are used to investigate the effect of electric field on the aptamer-thrombin complex; the computational domains of these two approaches are marked out in Fig. 1(b). We find that for positive electric fields the thrombin separates from the aptamer facilitated by a reduced free energy of binding.

## Results and Discussion

### Electric field inside the DNA layer

The electric field variation with distance from the electrode surface as obtained from the solutions of the PBE described in equation (3) are plotted in Fig. 2(a,b) for grafting density *σ* = 10^12^ cm^−2^ and 10^11^ cm^−2^ respectively. We observe from Fig. 2(a) that initially the field is constant up to 1 nm from the electrode surface as this space is occupied by the neutral portion (thiol) of the linker. Further away, the field varies linearly as the DNA charge is constant within the layer. The change in the field is more pronounced inside the region occupied by TBA G-quadruplex (between 15 and 16.6 nm) as evidenced by the steeper slope. Here, the occurrence of stronger charge density relative to the rest of the DNA molecule produces a significant change in electric field over a shorter distance. Outside the layer the field lines are nonlinear because of the hyperbolic charge density of the ions. We find from Fig. 2(a) that the field inside the DNA layer becomes progressively positive with increasing positive electrode potentials. At an electrode potential of (+) 300 mV the field, inside the DNA layer, is predominantly positive relative to that for (−) 300 mV. Similar characteristics are observed for the electric field for a reduced DNA density in Fig. 2(b), but the field is entirely positive over the whole domain for positive electrode potentials. Figure 2(a) also reveals that at *σ* = 10^12^ cm^−2^ and for an electrode potential of (+) 300 mV, the electric field inside the DNA layer switches from positive to negative across the layer height through a change in magnitude of ~0.06 V nm^−1^. In contrast, from Fig. 2(b) it is found that for *σ* = 10^11^ cm^−2^. while the electric field remains positive inside the layer, there is a slight drop in the field strength across the layer thickness even if it is an order of magnitude lower at ~0.002 V nm^−1^. Expectedly the more negative charges on account of the denser DNA layer produce enhanced shielding even when the electrode potential is substantially positive, thus explaining the greater drop in field strength for the higher grafting density. Hence, our results show that the electric field distribution in the negatively charged layer of DNA depends on the grafting density and applied surface potentials. For *σ* = 10^12^ cm^−2^ the electric field near the surface of the DNA layer, where the aptamer head containing the G-quadruplex is located, remains negative for the range of potentials investigated in this study. At the lower grafting density of *σ* = 10^11^ cm^−2^, the electric field across the aptamer head is entirely positive for all three of the positive potentials investigated in this study. So under suitable electrode potential and grafting density, the field generated by the negatively charged DNA layer can be turned to positive. Physiologically, thrombin is positively charged^12^ and, hence, would be repelled by the positive electric field inside the DNA layer. Therefore, a positively charged molecule situated over a TBA layer at an electrode potential of (+) 300 mV will experience a repulsive force that is stronger compared to that at (+) 100 mV since the electric field becomes increasingly positive with increasing positive electrode potential. For a potential of (−) 300 mV, the magnitude of the field at the top of the DNA layer is more negative for *σ* = 10^12^ cm^−2^ compared to that for *σ* = 10^11^ cm^−2^. In both cases, thrombin would be attracted towards the layer, as shown in earlier experiments^23^. One of the assumptions behind the continuum analysis is that the structure of the TBA is maintained on binding with thrombin which is based on observations that the G-quadraplex structure of TBA is stabilized on binding with thrombin protein^24^. Also folding of the 35-mer linker as it is condensed upon itself may be approximated by decreasing the aptamer layer height. Continuum calculations for grafting density of *σ* = 10^11^ cm^−2^ as well as *σ* = 10^12^ cm^−2^ show that decreasing the height of linker layer results in similar trend in dependence of electrical fields on applied potential as shown in Fig. 2a and b. Decreasing the linker layer height makes the electrical field at the top of the layer increasingly positive (explained in detail in Supplementary information S1). Hence, the electrical filed magnitudes plotted in Fig. 2 correspond to lower approximation for the electrical fields. Thus we conclude that the strength of this positive field increases with an increase in the applied positive electrode potential, increased condensation of the linker portion of the aptamer strand and reduction of grafting density.

### Effect of electric field on TBA/thrombin complex

**Figure 1:**
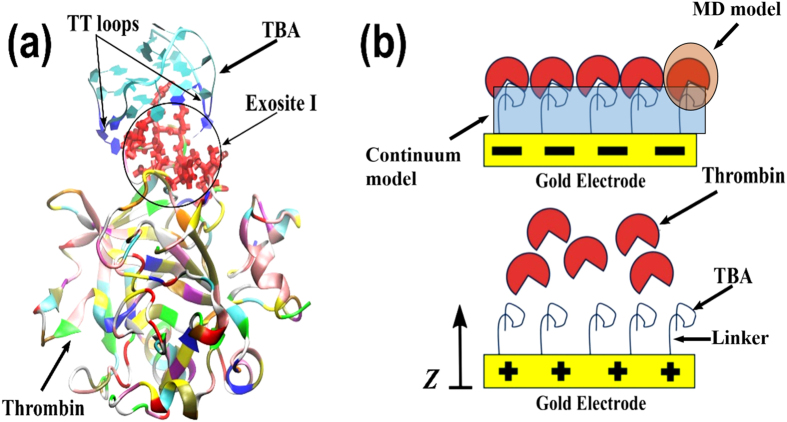
(**a**) Crystallographic structure (sourced from PDB entry 1HAO) of TBA/thrombin complex is presented by rendering in VMD^48^. The thymine residues (T3, T4, T12 and T13) of the TT loops of TBA are represented in blue whereas all the other DNA bases are shown in cyan. The exosite-I residues of thrombin are shown in red. (**b**) A cartoon describing the dissociation of TBA/thrombin complex on application of external stimulus^23^, as observed experimentally, is shown. The thrombin molecule is in red and the nucleic acid is represented by the stick with a coiled head representing the 15-mer TBA. The computational domains for the continuum as well as the MD simulations are also represented.

**Figure 2:**
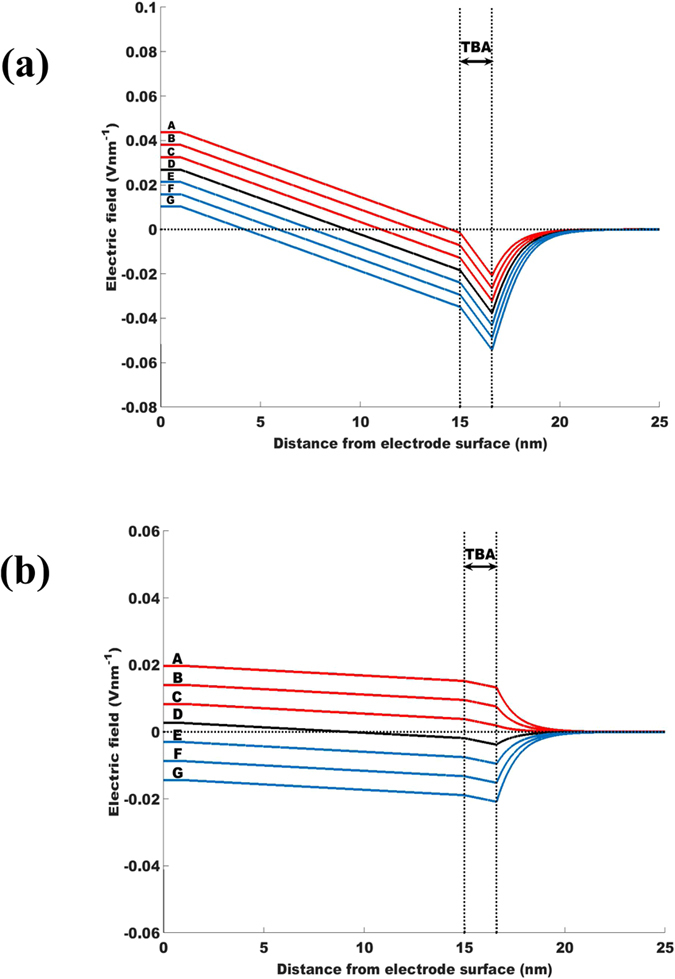
The evolution of the electric field from the electrode surface is presented for the TBA in salt solution, where the DNA density (**a**) *σ* = 10^12^ cm^−2^ and (**b**) *σ* = 10^11^ cm^−2^. Curves A, B, C, D, E, F and G respectively correspond to the electrode potential of +300 mV, +200 mV, +100 mV, 0 mV, −100 mV,−200 mV and −300 mV. The positive electrode potential is able to change the electric field inside the DNA layer from negative to positive and the change depends on the DNA density as well as the magnitude of the electrode potential. The 15-mer TBA occupies the domain between 15 to 16.6 nm, along the z-coordinate normal to the electrode surface, which is marked in the figure. Curves for the positive, neutral and negative electrode potentials are colored red, black and blue respectively.

**Figure 3:**
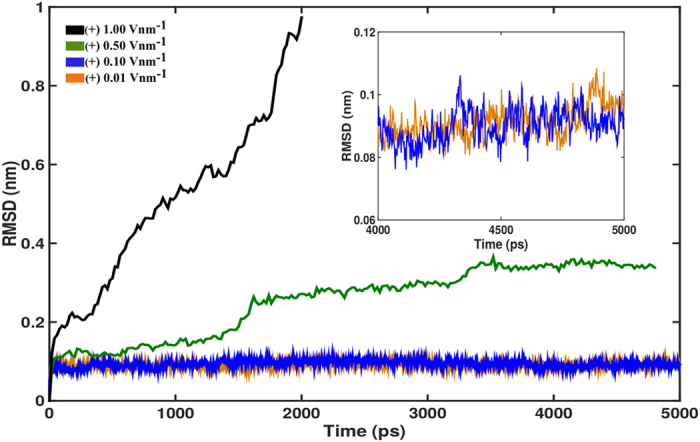
The transient evolution of thrombin RMSD for representative cases of electric field stimulus are shown as obtained from MD simulations. RMSD prediction is only for the time period when the thrombin is inside of the simulation box. An increase in the electric field magnitude results in an increase in the RMSD of the thrombin. Results of one representative simulated case from the 5 different sets of computations performed are presented here.

We examine the thrombin structure under different electric fields by employing MD simulations. We find that higher electric fields exerted a notable influence on the secondary structure of the protein. Root mean square deviation (RMSD) of the atomic positions of a protein obtained from simulation trajectories can be utilized to understand the conformational changes due to MD simulations of the protein with respect to a reference structure of the same protein at an equilibrated state. Analyses of RMSD of the thrombin molecule for different electric field strengths is shown in Fig. 3. The reference structure is chosen from the average structure of the predominant cluster (84% occupancy in the 50 ns trajectory) in the 50 ns production run and a backbone least square fit is performed for the electric field simulations spanning 5 ns. The results, in Fig. 3, show that for electric fields of (+) 0.01 and (+) 0.1 V nm^−1^ the structure is stable with an average RMSD value of around 0.085 nm. The inset shows the last 1 ns of the simulation for 0.01 and 0.1 V nm^−1^, where no deviations are noted from the average RMSD values. The RMSD curves for higher positive electric fields of (+) 0.5 and (+) 1.0 V nm^−1^ represent the dynamic behavior of thrombin structure. The RMSD for (+) 0.5 V nm^−1^ plateaus at ~0.32 nm after ~3500 ps (3.5 ns) of the simulation. We also note from Fig. 3 that under the electric field of (+) 1.0 V nm^−1^, the RMSD of thrombin increases very rapidly, indicating the stronger impact of the higher strength electric field on the protein structure and the thrombin molecule exits the simulation box within ~1.6 ns of the 5 ns simulation. Further analysis, on the thrombin structure under the influence of (+) 0.5 V nm^−1^, shows that the protein shape is conserved well with respect to the structure at the start of simulation (discussed in detail in Supplementary material S11, S12, S13, S14 and S15). Interestingly, thrombin spontaneously dissociates from TBA at (+) 0.5 V nm^−1^, in agreement with experimental findings that showed that the complex dissociated at an electrode potential of (+) 100 mV but remained bound at (−) 100 mV or the neutral condition (Fig. 1(b)) 23. This unbinding mechanism is illustrated in a video that is available as Supplementary material S1. This dissociation is not achieved under electric fields of (+) 0.01 and (+) 0.1 V nm^−1^ (Supplementary information S9). From the continuum analysis discussed previously (Fig. 2b), the top of the TBA layer is under an electric field of about (+) 0.006 V nm^−1^ and (+) 0.18 V nm^−1^, when the experimental electrode potential is (+) 100 mV and (+) 300 mV respectively. As in Supplementary information S1(i) and S1(j), if we only consider the 15-mer aptamer for the continuum modeling of the experiments^23^, then, at (+) 100 mV of electrode potential, for DNA grafting density of *σ* = 10^11^ cm^−2^ and *σ* = 10^12^ cm^−2^, the electric field at the end of the layer is roughly 0.04 V nm^−1^ and 0.03 V nm^−1^ respectively. However, in the MD simulations dissociation is achieved at around 10 times (0.5 V nm^−1^ as compared to 0.04 V nm^−1^) the electric field calculated in the continuum analysis. These differences can be attributed to the difference in time scales of the experiments, where the electrode potential is applied to the complex over a few minutes to observe the dissociation, and the atomistic simulations run for only a few nanoseconds. Earlier reports have shown that the use of larger electric fields in MD computations with relatively much shorter simulation times produce results comparable to experiments^21^,^25^.

The dissociation of thrombin is again observed on application of (+) 1.0 V nm^−1^, while for the simulations at (−) 0.1, (−) 0.5 and (−) 1.0 V nm^−1^, there is no unbinding of the complex. Strong electric fields can distort the protein and compromise its functions. Thrombin structure deteriorates rapidly, wherein the protein is unfolded and stretched, within 1 ns of the 5 ns simulation for an electric field of (±) 3.0 V nm^−1^ (snapshots of the simulation available as Supplementary information S10). The video of the simulation for the case of (−) 3.0 V nm^−1^, included in Supplementary material S2, displays that the thrombin remains bound with TBA even when the magnitude of the field is very strong and the protein structure is distorted. Earlier experiments have shown that application of voltages higher than (+) 300 mV can induce Faradaic currents on gold electrodes which in turn can denature DNA because pH changes can break gold-thiol bonds^26^. Thus, we conclude that successful modulation of the binding depends on the nature (positive/negative) as well as the magnitude of the electric field.

**Figure 4:**
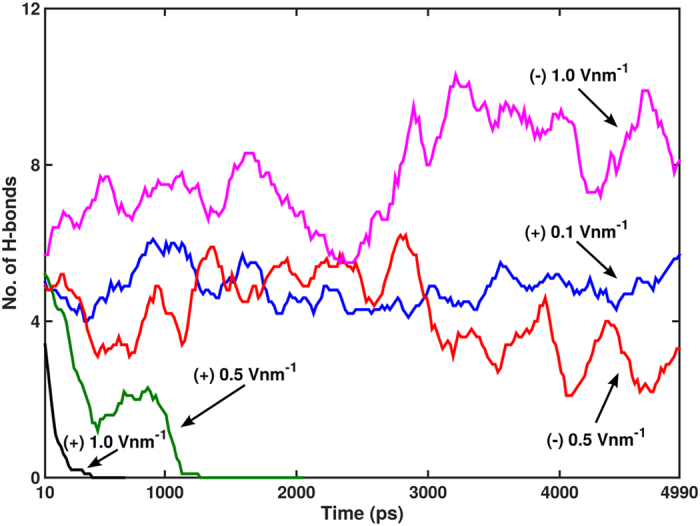
The H-bond population between TBA and thrombin for representative cases of electric field stimulus are shown as obtained from MD simulations. A running average over 10 data points is used for clarity of presentation. As the electric field becomes progressively positive, the H-bonding between TBA and thrombin decreases. Results of one representative simulated case from the 5 different sets of computations performed are presented here.

A hydrogen bond (H-bond) analysis for representative MD simulations is presented in Fig. 4. The results imply a direct relation between electric field strength and H-bond formation. H-bonds cease to exist as the complex gets separated and the rate of decrease is directly proportional to the increasing positive electric field. Under the electric fields of (+) 0.5 and (+) 1.0 V nm^−1^, thrombin is dissociated from TBA and exits the simulation box at ~2157 ps and 790 ps, and H-bonds between the two molecules do not form beyond ~1220 ps and 390 ps, respectively. We determine the existence of an interaction between specific residues of the protein and the aptamer by analyzing the hydrophilic interactions, in terms of H-bonds, as well as the hydrophobic contacts between the protein amino acid side chains and the DNA bases. Previous efforts have shown that Arg-75 of thrombin forms hydrogen bonds with the T13 and T4 residues of the aptamer and Arg-77A interacts with T13 and G14 bases^17^,^27^. These interactions are also observed in the simulations with positive electric fields, but their formations are notably reduced. Instead, the T4 base is found to interact with Arg-77A during the dissociation process. It is observed that both NH1 and NH2 atoms of Arg-77A donate a hydrogen to one of the carbonyl O atoms of the T4 base as thrombin gradually unbinds from TBA. An important interaction not present in the simulations with (+) 0.5 and (+) 1.0 V nm^−1^ is the H-bond between Tyr-76 and T4, as detailed in previous literature^17^,^27^, although it is observed for the smaller positive electric field of (+) 0.1 V nm^−1^. H-bond count at the start of simulation is relatively lower for the higher positive electric fields. We also note from Fig. 4 that for the last 2000 ps of the simulation the average H-bond count is less for the (−) 0.5 V nm^−1^ field relative to that for the (+) 0.1 V nm^−1^. Visual analysis of the simulation for (−) 0.5 V nm^−1^ reveals that thrombin is still bound to the TBA. Also the globular protein undergoes an overall angular movement wherein it is shifted along the *y*- axis of the simulation box. Further analysis shows that the exosite-I residues have limited avenues to interact with the TT loops of the aptamer, due to the reorientation of the protein. A new interaction between Lys-110 and T3 is observed; the NZ group donates hydrogen to the N3 and later to the O2 group of atoms in the T3 base of TBA, during the course of the simulation. As the electric field is increased to (−) 1.0 V nm^−1^ it is observed that the thrombin is pushed backwards (backward drift) along the + z axis towards the TBA. This facilitates additional interatomic interactions, which in turn results in a considerable increase in H-bond count when compared to that observed for other electric field simulations. The T3 base is observed to participate in H-bonding with Met-84, Arg-67, Lys-81 and Lys-110 residues of thrombin. Also prominent interactions are observed between Arg-73 and G6, Ser-72, Thr-74 and Arg-67 with G5, Arg-77A, Lys-109 and Arg-67 with G2. The interactions with the G-bases of the aptamer are enabled by the reorientation of the protein due to the backward drift under the negative electric field. Thus we find that under the negative electric fields the protein reorients with respect to the aptamer, newer interactions are promoted and the complex is still bound (Supplementary information S16).

### Insight from SMD simulations at (−) 1.0 V nm^−1^

**Figure 5:**
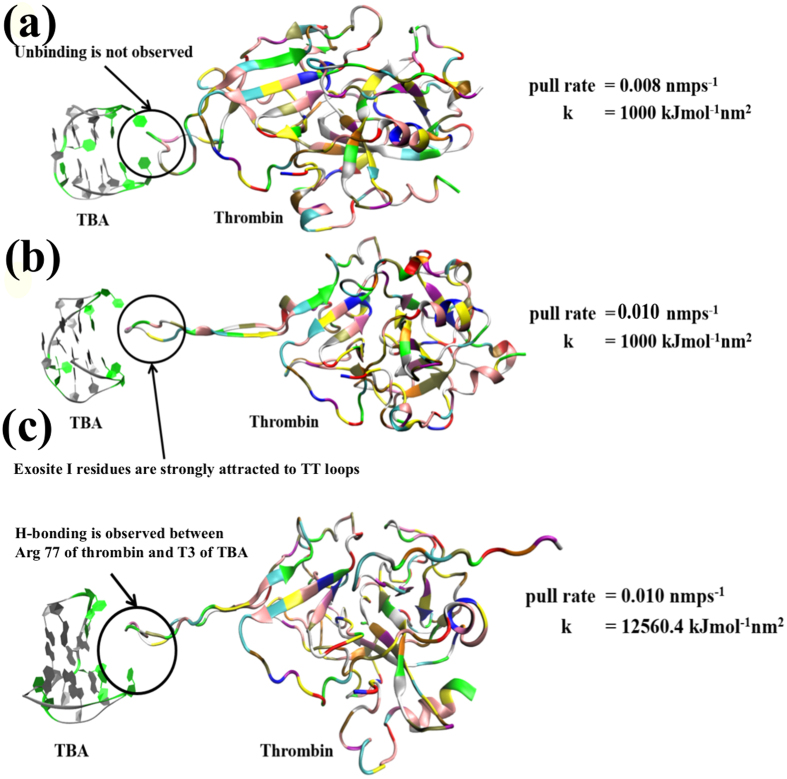
The binding between thrombin and TBA is shown, as observed in different SMD simulations, with varying force constants and pull rates, for the electric field of (−) 1.0 V nm^−1^ and (**a**) pull rate = 0.008 nm ps^−1^, harmonic constant = 1000 kJ mol^−1^ nm^2^, (**b**) pull rate = 0.01 nm ps^−1^, harmonic constant = 1000 kJ mol^−1^ nm^2^, (**c**) pull rate = 0.01 nm ps^−1^, harmonic constant = 12560.4 kJ mol^−1^ nm^2^. At (−) 1.0 V nm^−1^, there is a backward drift on the thrombin which promotes stronger binding with the TBA and thus a greater pulling force is required to unbind the complex. All images are rendered in VMD^48^.

The results of the pulling simulation at (−) 1.0 V nm^−1^ are presented in Fig. 5. Figure 5(a) and (b) show that complete dissociation of thrombin from TBA is not achieved for pull rates of 0.008 and 0.01 nm ps^−1^ when using a harmonic force constant of 1000 kJ mol^−1^ nm^2^. Even upon increasing the force constant to 12560.4 kJ mol^−1^ nm^2^ (Fig. 5(c)), as employed in the literature^22^, we find that complete unbinding does not occur. Extensive discussions on the choice of the restraining potential and its influence on the predicted results are provided in the supplementary document (S5). Experiments have demonstrated that at a negative electrode potential of (−) 300 mV there exists a pushing force which destroys the nanostructure of the complex upon the gold electrode^23^. H-bond analysis on the negative field SMD simulations prove that there is strong adherence of exosite-I residues to the TT loops as well as the G-quartet of TBA at (−) 1.0 V nm^−1^ even if a pull force is applied for breaking the bonds. As shown in Fig. 5(c), for the highest force constant as well as pull rate employed in our simulations, at (−) 1.0 V nm^−1^, there is strong interaction between the TT loops of TBA and exosite-I residues of thrombin. While at longer simulation times the bonds will eventually break under the effect of the pull force, the protein does experience a pushing force (opposite to the pull direction) under negative electric fields. As a result of this pushing force the thrombin experiences a backward drift i.e. opposite to the pull direction, towards the TBA, which in turn promotes the molecular interactions. This interpretation is supported by our results from the continuum analysis, as discussed above, that describes the electric field generated by the DNA layer at an electrode potential of (−) 300 mV to be primarily negative and affinity of the thrombin towards the TBA layer.

### Free energy of binding from SMD and US simulations

**Figure 6:**
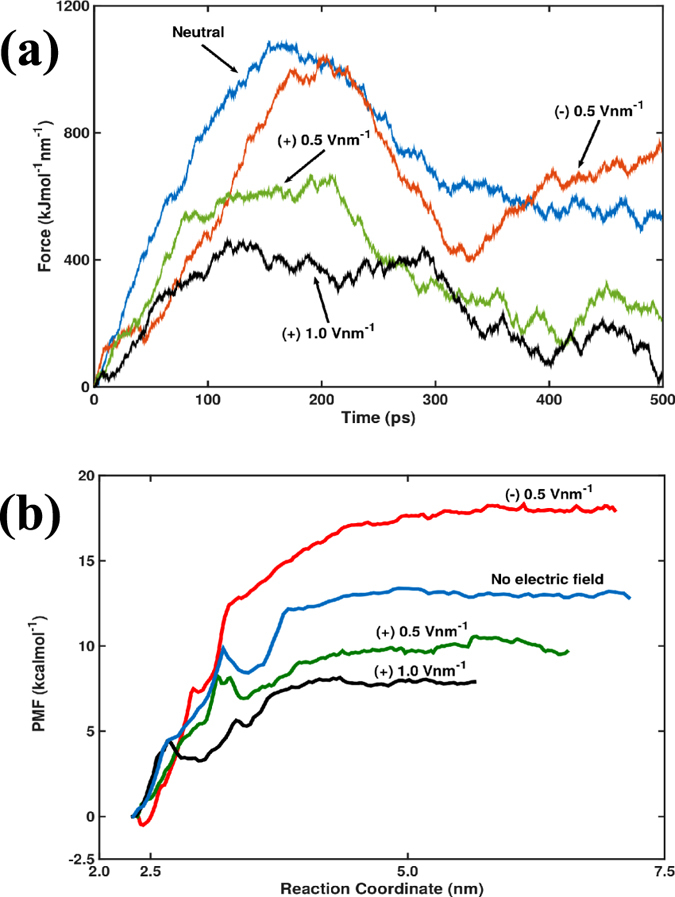
For representative cases of electric field stimulus in SMD simulations, the variation of (**a**) force (resulting from pulling the COM of thrombin) with time and (**b**) potential of mean force (PMF) evolution with reaction coordinate are presented. Positive electric fields lower the force on the thrombin molecule as well as the ΔG_binding_ of the TBA/thrombin complex. Results of one representative simulated case from the 5 different sets of computations performed are presented here.

Next, we calculate the free energy of binding between thrombin and TBA for specific electric fields of (+) 1.0 and (±) 0.5 V nm^−1^. Atomic trajectories are generated from SMD computations for use in US simulations. As described above, on pulling the COM of the protein, force builds up until a breaking point is reached when the critical interactions are disrupted, allowing the thrombin to dissociate from the aptamer. The stretching of the imaginary spring attached to the COM of the thrombin, upon the breaking of bonds within the TBA/thrombin complex, gives rise to the force. Figure 6A shows the evolution of this force with respect to the SMD simulation time for the (+) 0.5, (−) 0.5 and (+) 1.0 V nm^−1^ cases relative to that in absence of any electric field. For (+) 0.5 and (+) 1.0 V nm^−1^, the maximum force values are small because the application of these electric fields spontaneously dissociates the thrombin. For the neutral case with no electric field, the maximum force is ~1080 kJ mol^−1^ nm^1^ occurring at 168 ps at a COM separation distance of ~1.67 nm. The last hydrogen bond between TBA and thrombin is recorded at 238 ps at a COM separation distance of ~2.41 nm. The maximum force for (+) 0.5 V nm^−1^ is ~668 kJ mol^−1^ nm^1^ at 210 ps and for (+) 1.0 V nm^−1^ is 460 kJmol^−1^ nm^1^ at 117 ps, with the COM separation distances being 2.06 nm and 1.42 nm respectively. For (+) 0.5 V nm^−1^, the last hydrogen bond within the TBA/thrombin complex is noted at 210 ps. At the higher electric field of (+) 1.0 V nm^−1^ the last hydrogen bond breaks at 218 ps when a COM separation is 2.01 nm, but a second maximum value of the force (435 kJ mol^−1^ nm^1^) occurs at 289 ps. These results show that hydrogen bonding between thrombin and TBA ceases at lesser COM separation for increasing electric field. The reduction in H-bond formation within the TBA/thrombin complex on application of positive electric fields has been discussed previously in this article. The application of higher positive electric fields distort the protein, promoting unfolding and this exposes the hydrophobic core and facilitates some amount of hydrophobic interactions with the ligand. Even when the H-bonding ceased between the TBA and thrombin, at (+) 1.0 V nm^−1^, analysis revealed contacts between the hydrophobic residues Ile-82 and Ile-79 of exosite-I of thrombin and the T-3 and T-13 bases of TBA respectively. It may also be noted that there is absence of the gradual rise/decline of the force curve for the (+) 1.0 V nm^−1^ case as compared to the other cases. The hydrophobic interactions stymied the sharp descent normally observed in the force curves of SMD simulations. After the second maximum force point, further analysis did not reveal any interaction between TBA and thrombin. In the case of (−) 0.5 V nm^−1^ potential, the maximum force value is ~1041 kJ mol^−1^ nm^1^ recorded at ~204 ps at a COM separation of 2.10 nm while the last hydrogen bond is observed at 241 ps for a COM separation of 2.57 nm. The maximum force value for the negative field is greater than that for the positive fields but less than that predicted for the neutral case.

Table 1 lists the free energy of binding (ΔG_binding_) values calculated from the PMF (Fig. 6b) generated from the US simulations. We find that at (+) 0.5 and (+) 1.0 V nm^−1^, ΔG_binding_ is significantly reduced. This facilitates the spontaneous dissociation of thrombin from TBA on application of positive electric fields. The ΔG_binding_ for the (−) 0.5 V nm^−1^ case is greater than that for the neutral case even though the maximum force is relatively less. As the last H-bond as well as the maximum force occurs later in the dissociation path for the negative field compared to the neutral case, we conjecture that increased bonded/non-bonded interactions between thrombin and TBA result in a higher binding energy.

In all the COM pulling simulations, the point of maximum force corresponds to that instant when most of the bonds between the TT loop of the DNA and the interacting residues of exosite-I of thrombin are disrupted. The variations of the force on the COM of the thrombin with time derived from the SMD simulations are not sufficient to characterize the unbinding of TBA/-thrombin complex as this dissociation depends on the direction of pulling, choice of COM and the external applied potential. For a consistent COM definition and pulling direction, the dissociation depends on the events leading up to the separation between protein and DNA as well as the sign and magnitude of the electric field. If the dissociation path is similar, then a quantitative analysis of the unbinding of two molecules can be provided based on the transient evolution of the pull force. In all our SMD simulations the direction of pulling is always along the +*z* direction and this constrains the dissociation route. We find that for the positive fields the occurrence of maximum force (considering the 2^nd^ maximum for (+) 1.0 V nm^−1^ case as the 1^st^ and 2^nd^ maximum are close in value) is delayed in time relative to the neutral case. We attribute this to the slower pulling rates resulting in a prolonged dissociation event and lesser COM separation distance. This prediction is validated from the PMF profile in Fig. 6(b), which shows that the reaction coordinate sampled is lesser for these electric fields. For the negative electric field, there is a backward drift (explained previously in the article) on the thrombin molecule that delays the occurrence of the maximum force compared to the neutral case. This backward drift causes an increase in the pull force on the thrombin at the end of the simulation after the drop in the force between 200 and 300 ps.

**Figure 7:**
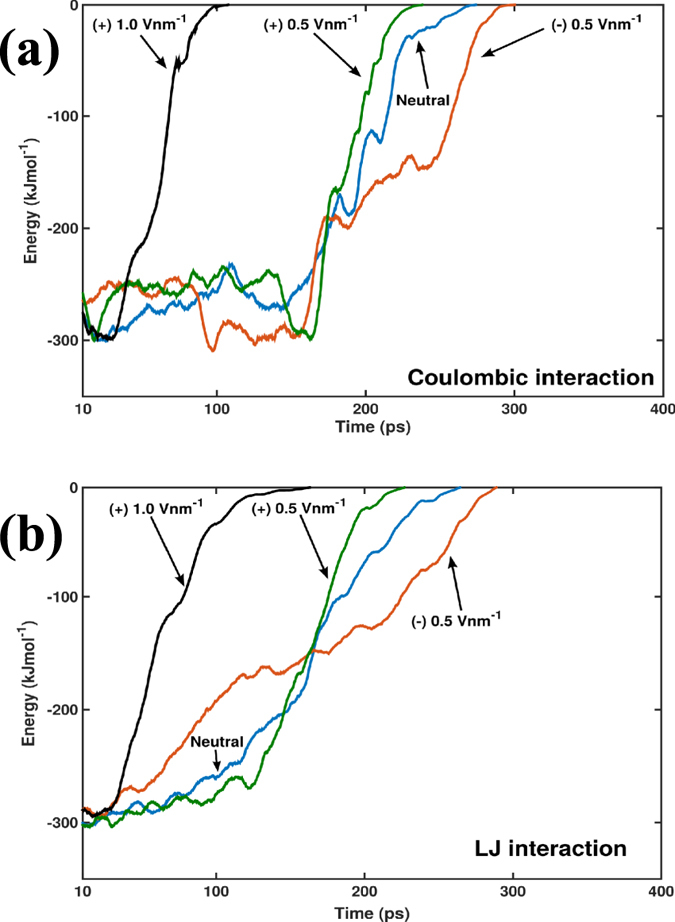
The transient evolution of the (**a**) electrostatic Coulombic and (**b**) potential energies due to non-bonded intermolecular interactions between the TBA and the thrombin molecules are shown for representative electric field SMD simulations. A 10 point running average of the data is illustrated for clarity of presentation. Up to 400 ps of simulation time is shown as the energies reach zero value due to separation of TBA/thrombin complex and a shorter range along the *x*-axis facilitates in distinguishing the curves. With progressive change of the applied electric field from negative to positive, the sum total of the non-bonded interaction energies are also reduced. The analysis of the non-bonded energies explain how the applied electric fields influence the energy landscape of the biomolecular complex, favoring or opposing the dissociation as suggested by the ΔG_binding_ for the different cases considered. Results of one representative simulated case from the 5 different sets of computations performed are presented here.

The electrostatic Coulombic and the Lennard-Jones (LJ) energies between the TBA and the thrombin molecules are monitored during the SMD simulations and are shown in Fig. 7(a) and (b) respectively. The Coulombic energy gives a measure of the extent of electrostatic interactions between the TBA and thrombin while the LJ energies quantify the dispersion/attractive as well as the repulsive/van der Waals interactions. From Figure 7(a) and (b) we find that both the Coulombic as well as the LJ interaction energy progressively increases for electric fields of (+) 1.0, (+) 0.5, 0 and (−) 0.5 V nm^−1^. We conclude that the higher positive electric fields decrease the interaction energy between TBA and thrombin resulting in lower ΔG_binding_. For the (+) 1.0 V nm^−1^ case, the increments (from negative to zero) in both the Coulombic and LJ interaction energies are rapid, as noted by the steeper curves over a short time interval, relative to the (+) 0.5 V nm^−1^ case. Consequently, ΔG_binding_ is smaller for (+) 1.0 than that for (+) 0.5 V nm^−1^. On the other hand the electric field of (−) 0.5 V nm^−1^, shows enhanced Coulombic interactions accompanied by higher LJ interaction energies. For the following representative electrical fields, (+) 1.0, (+) 0.5, 0 and (−) 0.5 V nm^−1^, the total electrostatic Coulombic energies are −1.68, −4.82, −5.48 and −6.02 × 10^5^ kJ mol^−1^ and the total LJ interaction energies are −1.84. −4.53. −4.68 and −4.97 × 10^5^ kJ mol^−1^ respectively. Expectedly the negative field case has a higher PMF value compared to that of the neutral case.

## Conclusion

We have employed a combination of continuum model and high-performance molecular computations to understand the role of external electrical stimulus on the binding and dissociation of thrombin-TBA complex. The simple, yet powerful, Poisson-Boltzmann theory based on a mean field model of the electrostatic interaction between particles reveals that at positive electrode potentials the DNA layer forms a repulsive region for the thrombin molecule, thereby acting as a deterrent to the complex formation. Predictions of the free energy of binding for the complex for different electric fields obtained from umbrella sampling simulations also suggest that for positive electric fields, the ΔG_binding_ is lesser compared to that of the neutral/negative case. The electrical stimulus influences the non-bonded interaction energies between thrombin and its aptamer as well as the H-bonding between the two; positive electric field reduces the non-bonded interaction energy and H-bonding between the TBA/aptamer complex which results in a reduced ΔG_binding_. In conjunction with earlier experiments, our molecular dynamics simulations of the TBA/thrombin complex under positive electric fields show that the complex dissociates spontaneously and that the binding between protein-ligand complexes can be controlled through electrical stimulus. Our results open up an exciting avenue for potential biomedical applications involving DNA/protein complexes. The electrical field mediated modulation technique can be extended to processes involving targeted or controlled release of drug by application of external stimulus.

## Methods

### Continuum modeling of the TBA layer

Single strand (ss) DNA can be effectively modeled by the polyelectrolyte brush theory for sufficiently high (~10^12^/nm^2^) electrode grafting densities, wherein, the DNA strands are assumed to be stretched forming rod like structures. The charges due to the phosphate groups on the DNA bases are spaced in 0.4 nm intervals^7^. For the continuum analysis the TBA layer is assumed to be in a 0.1 M salt solution and we define a spatial direction *z* normal to the electrode surface (Fig. 1(b)). The thrombin molecule is not included in this analysis. The DNA used in previous experimental work^23^, consisted of the 15-mer TBA along with a 35 base long tail and a thiol end, forming the linker with the gold electrode (Fig. 1(b)). The TBA has a G-quadraplex configuration resembling an armchair^28^, as shown in Fig. 1(a), hence, we approximate the charge distribution in DNA brushes with lower charge density over the height of 35-mer linker and higher charge density over the TBA layer. Initial calculations are done assuming the linker and TBA layer follow a rod-like structure. The effect of linker DNA condensation or folding is approximated by assuming lower heights for linker layers (Supplementary information S1). The charge due to the 15 bases is uniformly distributed along the axial length of the TBA molecule (1.6 nm) as calculated from the structure in protein data bank (PDB) entry148D^28^. We also take into account the renormalization of the backbone negative charges of the DNA due to counterion condensation as described by Manning^29^.

The charge density of the TBA is expressed as


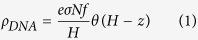


where, *e* is the electron charge, *f* the charging fraction calculated using Manning condensation theory, *N* the number of DNA bases, *H* the DNA layer height and *θ* is the Heaviside step function. The aptamer sequence used in the experiments has, 35 nucleotides in the long tail and 15 nucleotides in the aptamer head. The charging fraction is calculated as *f* = *b*/*l*_*B*_, where the Bjerrum length *l*_*B*_ = *e*^*2*^/*εk*_*B*_*T*, where *ε* is relative permittivity of the medium, *b* the separation per charge, *k*_*B*_ the Boltzmann constant and the temperature, *T* = 300 K. For our system, *b* = 0.4 nm and *l*_*B*_ ~ 0.7 nm, and hence *f* = 0.57. A 1 nm long charge neutral thiol linker molecule is also included in the model to mimic the thiol modification used to immobilize the aptamer with gold/thiol bond^30^.

The counterion charge density is given by a modified Poisson-Boltzmann equation described by Borukhov *et al*. considering the finite size of ions^31^:


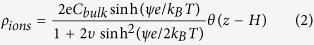


where, *C*_*bulk*_ is the bulk counterion concentration, *k*_*B*_ the Boltzmann constant, *ψ* the electrostatic potential of the system, *ν* the steric parameter limiting the ion concentration. *ν* = 2 C_bulk_ × (ion radius)^3^,^31^. The ion radius is 1.96 × 10^–7^ cm^7^. For the analysis counterions are excluded from the DNA layer as this provides the best agreement with experiments^7^. Equations (1) and (2) are used in the Poisson-Boltzmann equation and non-dimensionalized to obtain the second order differential equation governing the system:


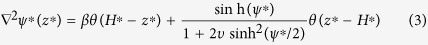


where ψ* is the nondimensional electrostatic potential and H* the non- dimensional DNA layer height. The electrostatic potential, *ψ*, and the spatial domain *z*, are non-dimensionalized by (*k*_*B*_*T*/*e*) and the Debye length λ_D_ = √(*8πl*_*B*_*C*_*bulk*_) respectively; the dimensionless coefficient *β* *=* *Nfσ*/*2HC*_*bulk*_. The governing equation is solved for boundary conditions of the external potential at electrode surface and zero electric field far away from the surface. The electrostatic potential distribution is computed for TBA layers with grafting densities varying from *σ* = 10^11^ cm^−2^ to *σ* = 10^12^ cm^−2^ consistent with the experimental efforts of our group^23^ and Stachowiak *et al*.^32^.

### Molecular dynamics simulation of thrombin-TBA unbinding

The crystallographic structure of TBA/thrombin complex (Fig. 1(a)) is obtained from the PDB entry 1HAO^33^. A combination of the force fields AMBER99SB, successfully employed to model proteins^34^, and parmbsc0, which has been shown to improve ss DNA MD simulations^35^, are used for our simulations on TBA/thrombin complexes^17^,^22^ in the GROMACS 4.6.7 package^36^. Following previously published literature^17^, the Asp, Glu and His residues of thrombin were protonated according to the discussion by Ahmed *et al*.^37^. The TIP3P water model^38^ is used to solvate the TBA/thrombin complex placed at the center of a rectangular 7.5 (*x*) nm × 7.5 (*y*) nm × 15 (*z*) nm) simulation box.; 26010 water molecules are added and 62 Na^+^ and 51 Cl^−^ ions are included in the system to neutralize biomolecular charge and build up 0.1 M concentration.

The system is energy minimized using the steepest descent algorithm. Position restraints are applied to the TBA/thrombin complex and the system is equilibrated at 300 K and 1 bar with a time step of 2 fs (femtosecond) under (a) the canonical ensemble (NVT) using velocity rescaling thermostat^39^ with coupling time constant of 0.1 ps for 1 ns, followed by (b) another 1 ns simulation under the isothermal-isobaric ensemble (NPT) using the Berendsen barostat^40^ with coupling constant of 5.0 ps and the thermostat as before. The initial velocities are generated from a Maxwell distribution at 300 K at the start of the NVT equilibration. Subsequently, all position restraints are removed and a MD simulation is performed under the NPT ensemble for 50 ns employing the Nose-Hoover thermostat^41^,^42^ with a coupling constant of 0.5 ps and the Parinello-Rahman barostat with coupling constant of 1.0 ps. In all the MD simulations the covalent bonds are constrained by the LINCS algorithm^43^ and periodic boundary conditions are applied in all directions. All non-bonded interactions are cut off at 1.4 nm and an FFT grid density of 0.12 nm is used with the particle mesh Ewald (PME) method^44^. Atomic trajectories and thermodynamic data are recorded every 0.1 ps. Average structure of the thrombin-aptamer complex from the trajectory of the 50 ns production run is used for electric field MD simulations, where a time invariant constant magnitude electric field is applied along the (+) *z* axis. The TBA is position restrained to the energy minimized configurations as experimental results have shown that binding with thrombin protein stabilizes the G-quadraplex structure of the aptamer. Production simulations under the NPT ensemble spanning 5 ns are carried out to investigate the configurational changes of thrombin in presence of the different applied electric fields: (±) 0.01, (±) 0.05,(±)1.0 V nm^−1^ and (±) 3.0 V nm^−1^.

Steered molecular dynamics (SMD) simulations^22^,^45^ are executed on the average complex structure obtained from the 50 ns production simulation for 500 ps using a pull rate of 0.01 nm ps^−1^ and a force constant of 1000 kJ mol^−1^ nm^2^. Here, the TBA is position restrained, the z axis is chosen as the reaction coordinate and the center of mass (COM) pulling technique is implemented, wherein the thrombin is pulled along the reaction co-ordinate. The free energy of binding (ΔG_binding_) between TBA and thrombin molecule is calculated using the umbrella sampling (US) technique and the WHAM (Weighted Histogram Analysis Method) algorithm^45^,^46^,^47^. 42 windows are selected for the umbrella sampling simulations from a series of configurations extracted from the trajectory of the SMD simulation. In each of these windows a short 100 ps NPT equilibration followed by a 10 ns umbrella sampling simulation under the NPT ensemble is undertaken for a total of 424.2 ns of umbrella sampling simulations. The results are incorporated into the WHAM framework to compute the potential of mean force (PMF) profile (more details provided in Supplementary information S5 and S17).

SMD simulations for 500 ps are also performed under influence of (±) 1.0 V nm^−1^ and (±) 0.5 V nm^−1^ electric fields along the (+) *z* axis. Pull rates of 0.006 nm ps^−1^ and 0.008 nm ps^−1^ are respectively used for (+) 1.0 V nm^−1^ and (+) 0.5 V nm^−1^ cases to ensure full dissociation of the complex,while for all other simulations 0.01 nm ps^−1^ is sufficient. Each of the 5 ns electric field MD and 500 ps SMD simulations were conducted independently for 5 times to observe the average behavior of the system. Additional details on the MD simulations are included in the supplementary information (S4, S5, S6, and S7).

## Additional Information

**How to cite this article**: Gosai, A. *et al*. Electrical Stimulus Controlled Binding/Unbinding of Human Thrombin-Aptamer Complex. *Sci. Rep.* **6**, 37449; doi: 10.1038/srep37449 (2016).

**Publisher's note:** Springer Nature remains neutral with regard to jurisdictional claims in published maps and institutional affiliations.

## Supplementary Material

Supplementary Video 1

Supplementary Video 2

Supplementary Information

## Figures and Tables

**Table 1:** The free energy of binding (ΔG_binding_) for the different umbrella sampling simulation cases are presented.

Electric field (V nm^−1^)	0	(+) 0.5	(+) 1.0	(−) 0.5
ΔG_binding_ (kcal mol^−1^)	12.5 ± 0.9*	8.5 ± 0.5	7.5 ± 0.7	17.5 ± 0.5

The error for each case is calculated by the Bootstrapping method using the WHAM algorithm in GROMACS for 100 bins^46^.

^*^The corresponding predictions for no electric field case from earlier computational and experimental investigations are 17 kcal mol^−1^ 22 and 9 kcal mol^−1^ 27 respectively. The error values for each of the averages are calculated from the bootstrap sampling method explained in Supplementary information S17 (b).
