# Supplementary material for: Electrical Stimulus Controlled Binding/Unbinding of Human Thrombin-Aptamer Complex
Source: Sci Rep. 2016 Nov 22;6:37449. doi: 10.1038/srep37449 (PMC5118750; doi:10.1038/srep37449)
Supplement: Supplementary Information [file srep37449-s3.pdf]

Electrical Stimulus Controlled Binding/Unbinding of Human Thrombin-Aptamer Complex

Agnivo Gosai[a], Xiao Ma[a],Ganesh Balasubramanian[a,b] and Pranav Shrotriya[a,1]

[a] Department of Mechanical Engineering and [b] Microelectronics Research Center, Iowa State University, Ames, IA 50011, USA

Supplementary Information

S1: Effect of DNA layer condensation on the electrical field near the gold surface.

We approximated the effect of the condensation or folding of DNA layer using two assumptions: (1) the 15-mer TBA retain their G-quadraplex structure since the aptamer structure is stabilized on binding with thrombin protein; and (2) The charge distribution of the 35-mer linker layer is distributed over shorter distance as the linker chains fold near the surface. The calculations are performed for the different densities of DNA layers namely, = 10^{11} and 10^{12} cm^{-2}. These results show that for all the surface densities, the trends of electrical field dependence on the surface potential remain the same but the magnitudes of electrical fields increase with chain folding or condensations. Thus the results with rod-like fully stretched chains provide us with lowest estimate for the field magnitude.

[1] Corresponding author
Address: 2019 Black Engineering Building, Ames, IA 50011
Phone: 515-294-9719, Email: shrotriy@iastate.edu

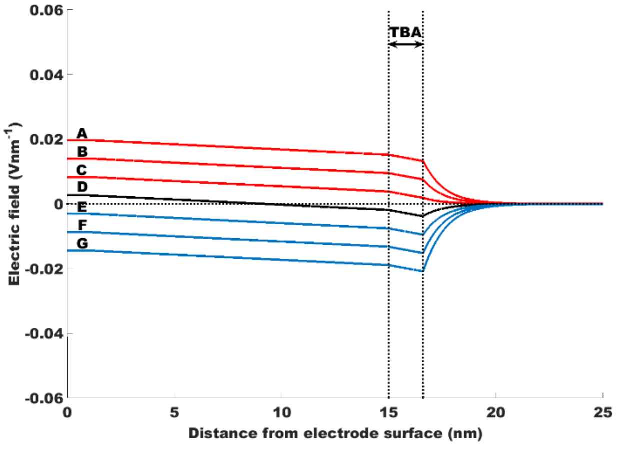

Figure S1 (a)

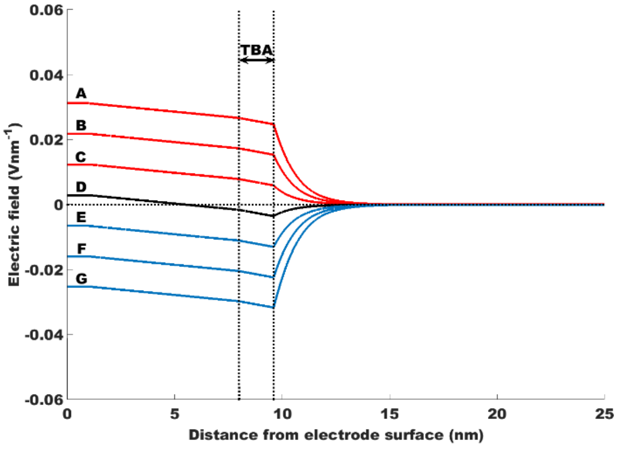

Figure S1 (b)

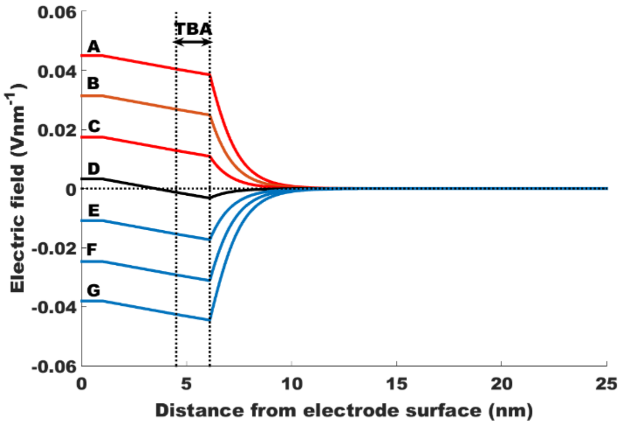

Figure S1 (c)

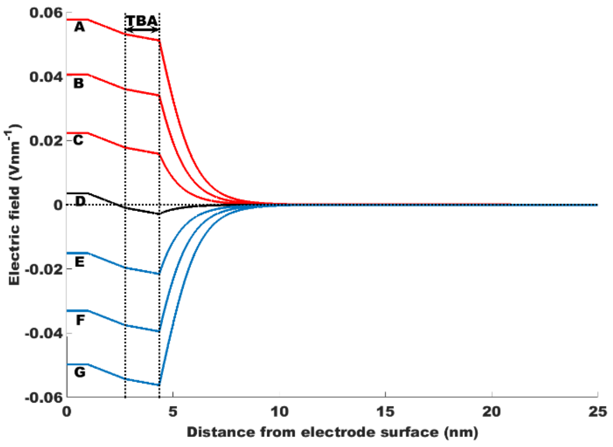

Figure S1 (d)

We consider the case where the nucleic acid grafting density is $\sigma = 10^{11}$ cm^{-2}. In the main article, Figure 2b corresponds to a fully stretched nucleic acid. As the structure of TBA (15-mer aptamer) is maintained upon thrombin binding as well as in salt solution[1], we may look at cases where the 35-mer linker molecule (Figure 1(b)) is condensed upon itself, thus reducing the effective length of the structure. Figure S1 (a), S1 (b), S1 (c) and S1 (d) corresponds to the uncondensed/folded length and 1/2,1/4th and 1/8th of the original linker length. It is observed that for increasing positive electrode potentials the electric field at the top of the nucleic acid layer becomes more positive thus corroborating our experimental observations. Curves A, B, C, D, E, F and G respectively correspond to the electrode potential of +300 mV,+200 mV, +100 mV, 0 mV, -100 mV ,-200 mV and -300 mV .The domain occupied by the 15-mer TBA is marked in the figure. Curves for the positive, neutral and negative electrode potentials are colored red, black and blue respectively.

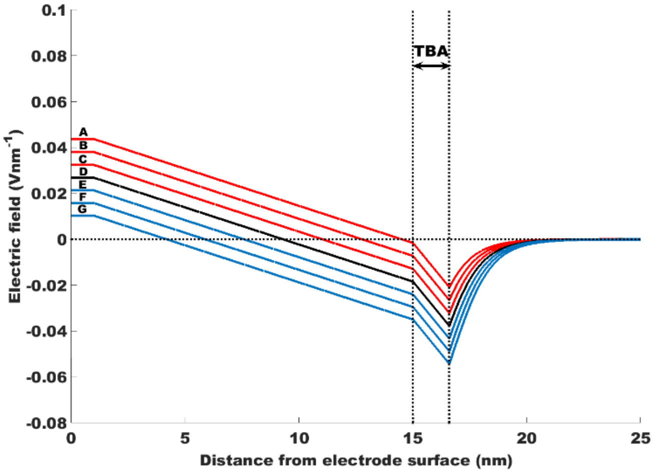

Figure S1 (e)

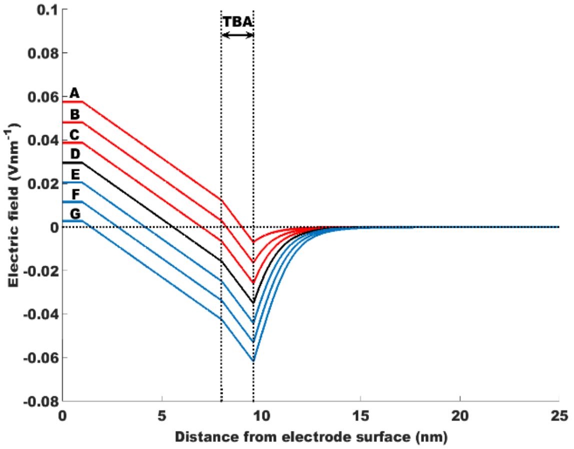

Figure S1 (f)

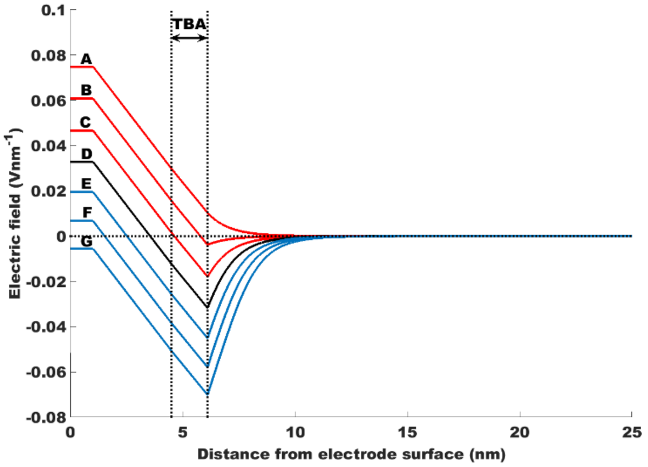

Figure S1 (g)

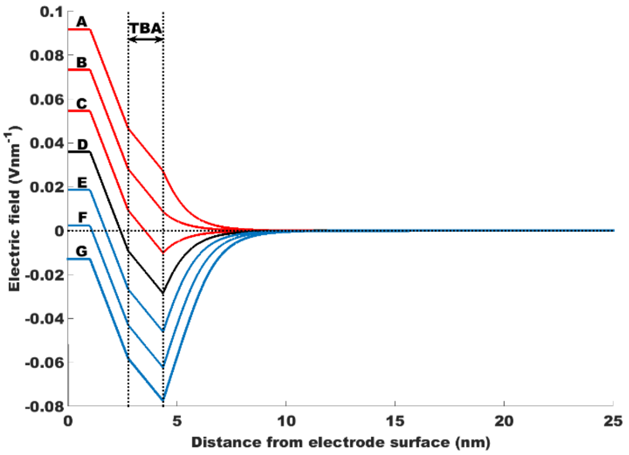

Figure S1 (h)

We consider the case where the nucleic acid grafting density is σ = 10^{12} cm^{-2}. Similar observations are observed as in the case discussed for σ = 10^{11} cm^{-2} . It is to be noted that on account of greater surface density of nucleic acid the positive nature of the electric field is more expressed for greater condensation of linker for the corresponding electrode potentials. Figure S1 (e), S1 (f), S1 (g) and S1 (h) corresponds to the uncondensed/folded length and 1/2, 1/4th and 1/8th of the original linker length. It is observed that for increasing positive electrode potentials the electric field at the top of the nucleic acid layer becomes more positive thus corroborating our experimental observations. Curves A, B, C, D, E, F and G respectively correspond to the electrode potential of +300 mV,+200 mV, +100 mV, 0 mV, -100 mV ,-200 mV and -300 mV .The domain occupied by the 15-mer TBA is marked in the figure. Curves for the positive, neutral and negative electrode potentials are colored red, black and blue respectively.

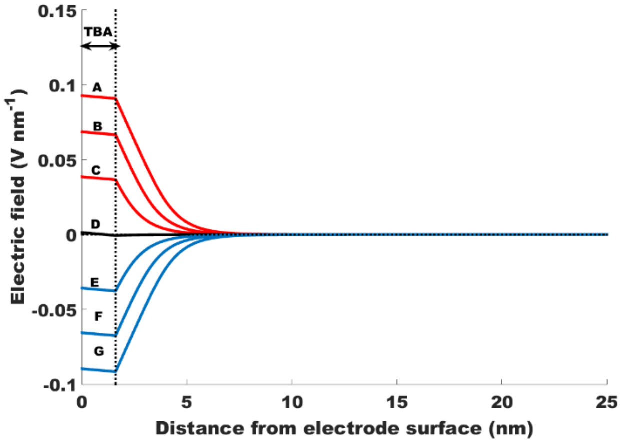

Figure S1 (i)

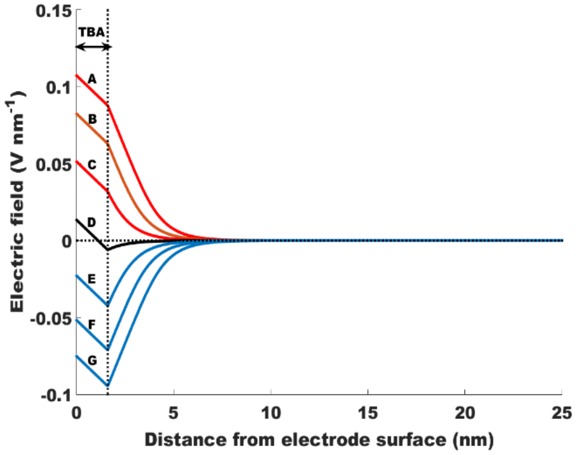

Figure S1 (j)

If we only consider, the 15-mer aptamer for the continuum modeling, we find that at (+) 100 mV for σ = 10^{11} cm^{-2}, the field at the top of the layer is ~ 0.04 Vnm^{-1} (Figure S1 (i)) and that for σ = 10^{12} cm^{-2} is ~ 0.03 Vnm^{-1}(Figure S1 (j)). This is thus an order of magnitude higher than the cases with the 35-mer linker molecule as discussed previously. In the MD simulations, only the 15-mer aptamer is included, and, the positive field at which dissociation is observed is about 10 times i.e. one order of magnitude higher than the electric field calculated from the continuum modeling. This difference is attributed to the large disparity in the time scale of the experiments and the atomistic simulations, as discussed in the main article.

In Figures S1(i) and S1(j) ,curves A, B, C, D, E, F and G respectively correspond to the electrode potential of +300 mV,+200 mV, +100 mV, 0 mV, -100 mV ,-200 mV and -300 mV .The domain occupied by the 15-mer TBA is marked in the figure. Curves for the positive, neutral and negative electrode potentials are colored red, black and blue respectively.

MD simulation video:

S2: The video shows the spontaneous dissociation of thrombin from TBA as observed in the MD simulation using the electric field of (+) 0.5 Vnm^{-1}. The TBA is position restrained throughout the simulation to mimic the experimental condition[2] where the aptamer is bound to the gold electrode surface through a thiol linker. 2 ns of a 5 ns simulation is shown only. Video is rendered using VMD[3].

S3: MD simulation video of TBA/thrombin complex under the influence of (-) 3.0 Vnm^{-1} electric field is presented. The video shows that thrombin is bound to TBA even if the protein gradually loses the secondary structure due to the effect of the high magnitude electric field. 3 ns of a 5 ns simulation is shown only. Video is rendered using VMD[3].

Note: The simulation data was collected every 0.1 ps of the simulation and during preparation of the videos one frame was generated every 1 ps.

S4: Choice of pulling velocities in electric field SMD simulations

Evidence in the literature suggest that the application of slower/faster pull rates result in almost identical trajectories and qualitatively similar force-time curves [4]. In practice, a convenient pulling rate is used to expedite data collection while maintaining the reliability of the procedure and ensuring that no artifacts are introduced in the results. We find that at high ((+) 1.0 and (+) 0.5 V nm^{-1}) positive electric fields using a pulling rate of 0.01 nm ps^{-1} resulted in the COM separation distance between the TBA and the thrombin to be greater than half of the box dimension along the z axis. This violates the convention for the periodic boundary condition of a rectangular simulation box. Hence, a relatively slower pull rate is employed. Supplementary Table 1 lists the details of the umbrella sampling simulations on the SMD trajectories for (±) 0.5 V nm^{-1} and (+) 1.0 V nm^{-1} cases.

Supplementary Table 1

Electric field (Vnm^{-1})	Umbrella sampling windows	Total simulation time (ns)	Pull rate (nm ps^{-1})	COM separation (nm)
**0**	**42**	**424.2**	**0.010**	**5.50**
**(+) 0.5**	**38**	**383.8**	**0.008**	**4.90**
**(+) 1.0**	**34**	**343.4**	**0.006**	**4.01**
**(-) 0.5**	**41**	**414.1**	**0.010**	**5.36**

S5: Choice of restraining potential for umbrella sampling:

Pagano et.al studied the thermodynamics of the interaction of TBA with thrombin through isothermal titration calorimetry (ITC) experiments[5]. They assumed a single set of equivalent binding sites to determine the binding constant from which the binding Gibbs energy change was calculated. At 25 ^{o}C , the $\Delta G_{binding}$ calculated by them was about 9 $kcalmol^{-1}$. The $\Delta G_{binding}$ calculated by Yang et al., from MD simulations, puts the value at 17 $kcalmol^{-1}$[6]. The ITC experiments did not permit a distinction between the two binding sites of thrombin as the titration data do not have distinct energy profiles and the value was measured based on the relative populations of the complex and non-complex forms. The Yang group argues that the $\Delta G_{binding}$ predicted by the MD simulations is larger than the experimental one because the simulation value is the free energy cost for a full separation of TBA from thrombin [6]. They also used a very high

force constant of 12560.4 $kJmol^{-1}nm^2$ and in their study the pulling coordinate is taken as the bond distance of the COM of TBA and the other end is the COM of the protein residues (Glu77-Lys81) enclosed by the TT loops of TBA. In the present study we instead take a much lower force constant of 1000 $kJmol^{-1}nm^2$ and our pulling coordinate is also different as described previously in the methodology section. We find that using a reduced force constant does not affect the convergence of the PMF. AFM pulling techniques have been utilized to unfold biomolecules like DNA hairpins to map the folding energy landscape by conducting equilibrium constant-force reversible folding-unfolding measurements. However AFM pulling experiments and data for TBA/thrombin complex, providing the $\Delta G_{binding}$, is still not available. From the theory of the umbrella sampling method it is understood that the ideal restraining potential would be the negative of the exact PMF, which is unknown for the present problem. When the negative of the exact PMF is used as the biasing potential it flattens the energy landscape and thus avoids the problem of getting trapped by large energy barriers. In absence of experimental data, researchers have used adaptive umbrella sampling method, multicanonical sampling method and the entropy sampling method, which unfortunately entail a lot of computational cost[7, 8].

All points along the reaction coordinate are to be sufficiently sampled for the WHAM method to produce a converged PMF. A necessary check for the convergence is the overlap and smoothness of the histograms generated from sampling of the reaction coordinate[9, 10]. The stipulation of this check ensures that all points along the reaction coordinate are thoroughly sampled in multiple simulation windows. These considerations determine the positioning of the umbrella sampling windows and the strength of the biasing potential i.e. the force constant. Proper sampling and thus the smoothness of the generated histograms also depends on the simulation time which is again constrained by computational costs. We have used an umbrella sampling simulation time of 10 ns per window against the 20 ns mentioned in the work by the Yang group[6]. We find that this does not greatly influence the smoothness of the umbrella sampling histograms. The histograms generated for the US simulations in the present study are shown in Fig. S9 (supplementary material). An optimum simulation framework that can minimize simulation cost and still preserve the accuracy of the generated results will greatly depend on the nature of the system on which the MD simulations are performed.

S6: Average over MD simulations

Each of the 5 ns electric field MD simulations and the 500 ps SMD simualtions were carried out 5 times to check the average behavior of the system under the different cases of electric field. The observations are represented in a tabular form as below:

Supplementary Table 2

**Electric field (Vnm^{-1})**	**Average H-bond count per frame of an 5 ns electric field MD simulation**					
	**#1**	**#2**	**#3**	**#4**	**#5**	**Average**
**(+) 0.1**	**5.54**	**5.31**	**5.78**	**5.82**	**5.45**	**5.58**
**(+) 0.5**	**1.48**	**1.37**	**1.68**	**0.98**	**1.52**	**1.41**
**(+) 1.0**	**0.48**	**0.91**	**1.29**	**0.68**	**0.72**	**0.82**
**(-) 0.5**	**3.26**	**2.53**	**3.35**	**3.49**	**3.16**	**3.16**
**(-) 1.0**	**7.65**	**7.16**	**7.70**	**7.78**	**7.71**	**7.60**

It is observed that with increasing positive electric field the H-bond count per simulation frame decreases whereas for the high negative field of (-) 1.0 Vnm^{-1} because of reorientation of protein and promotion of newer interactions the H-bond count increases. At (-) 0.5 Vnm^{-1}, the protein drifts away from the TBA and thus lesser H-bonds are formed towards the last part of the simulation.

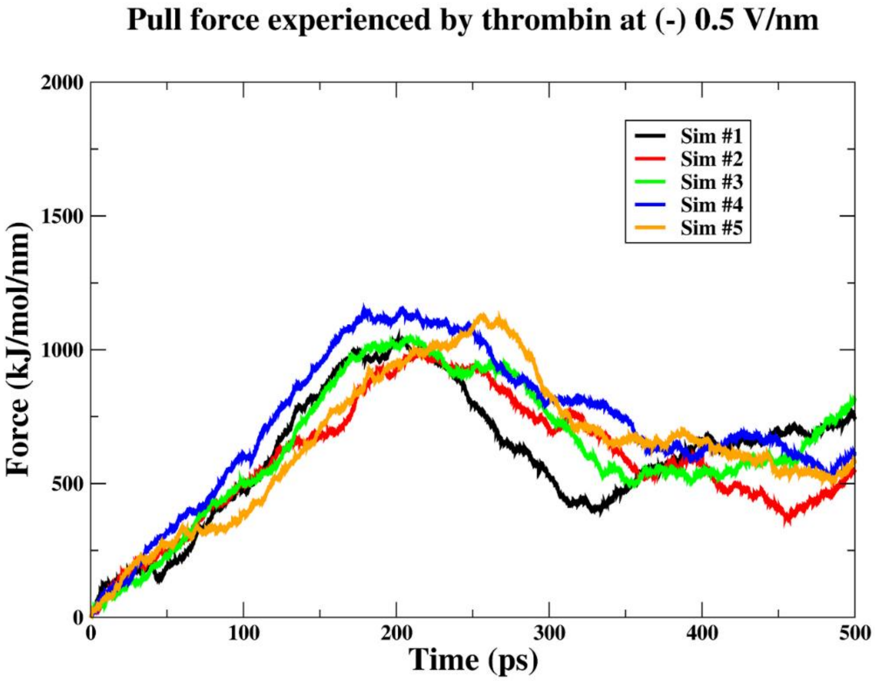

Figure S6

The above figure shows the pull force experienced by the COM of thrombin under the electric field of (-) 0.5 Vnm^{-1}. The 5 curves are from 5 different SMD simulations. The maximum force obtained from each of these 5 simulations are mentioned in Supplementary Table 3.

Supplementary Table 3

Electric field (Vnm^{-1})	Peak force obtained from a 500 ps SMD simulation (kJmol^{-1}nm^{-1})					
	#1	#2	#3	#4	#5	Average
0	1079.46	914.62	1120.32	1127.31	1053.71	1059.08
(+) 0.5	668.09	807.48	752.66	769.45	768.81	753.30
(+) 1.0	460.29	322.48	512.68	482.96	581.39	471.96
(-) 0.5	1041.32	996.95	1047.54	1154.89	1129.01	1073.94

We find that from the average values of the 5 simulations that the peak force experienced by the COM of the thrombin during the SMD simulations are progressively lesser in magnitude with increasing positive electric field. It is also noted that the peak force for the electric field of (-) 0.5 Vnm^{-1}, is somewhat higher than that observed in the neutral case.

S7: Exclusion of ligand OG6 from initial topology of MD simulations

The ligand OG6 was excluded from the coordinate of the PDB file 1HAO while preparing the topology of the MD simulation. The small molecule OG6 is an inhibitor called D-Phe-Pro-Arg-chloromethylketone (PPACK)[11]. It has got nonstandard residues which do not conform to the force field parameters used in the present study. OG6 is not associated with exosite-I of thrombin and the TBA/thrombin complex is found to be stable (without OG6) for long MD simualtions as described in figure S3. More details about OG6 are provided in the paper by Maitz et al. [11].

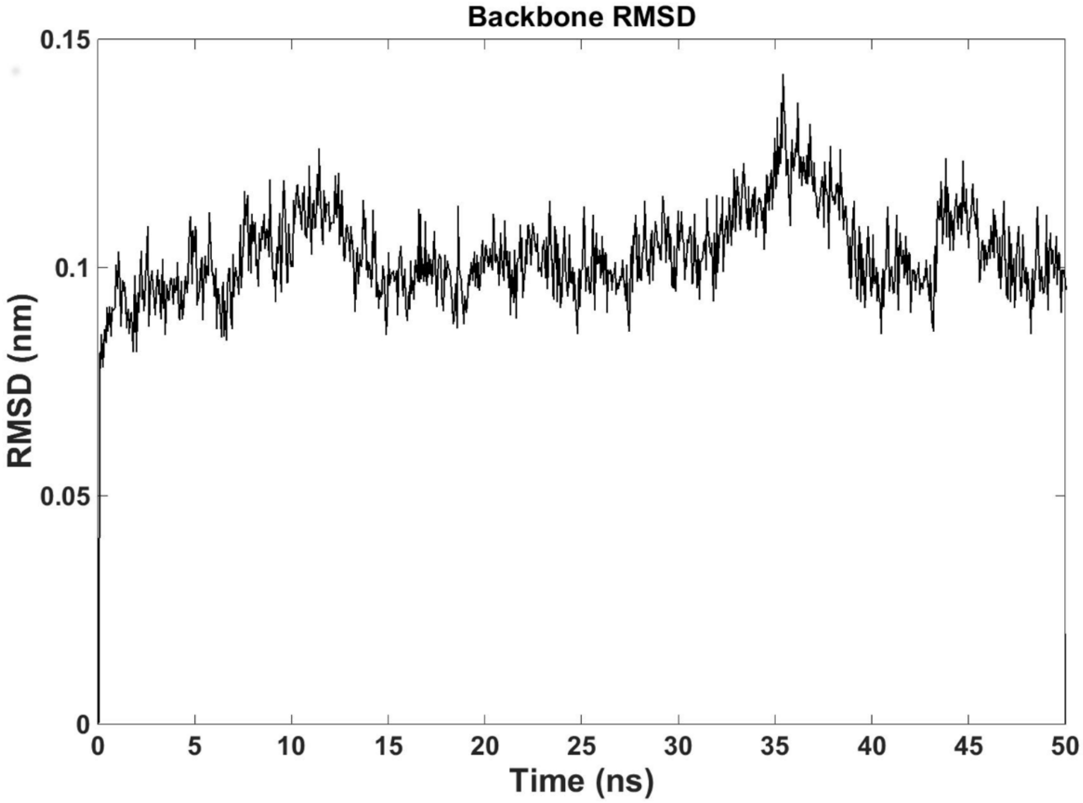

Figure S8

S8: The time evolution of backbone RMSD of thrombin, calculated with respect to the crystal structure, over the 50 ns unrestrained MD simulation is represented. The low average RMSD value of ~ 0.13 nm shows that the thrombin is stable over long simulation time. The ligand OG6 (used as an inhibitor for thrombin in laboratory applications) is excluded at the beginning of solvation as it does not interfere with the exosite-I of thrombin and the parameterization of non-standard residues could be avoided. However, it is imperative to check the stability of thrombin-aptamer complex on removal of a certain ligand molecule and hence the 50 ns simulation provided conclusive evidence for the same.

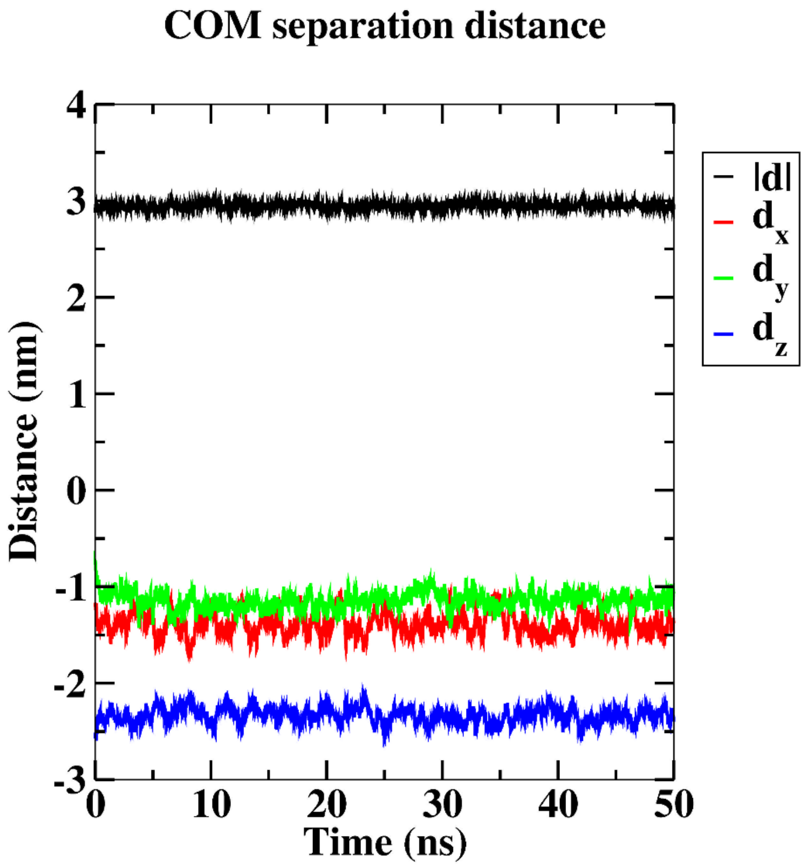

Figure S9

S9: The time evolution of the COM separation between TBA and thrombin is shown for a 50 ns MD simulation where the system was put under an **electric field of (+) 0.1 Vnm^{-1}**. The TBA is position restrained while the thrombin is free to move. However, it is observed that the COM separation (including the *x, y* and *z* components) is constant indicating that the TBA/thrombin complex did not dissociate. Same could be observed from the visualization of the simulation.

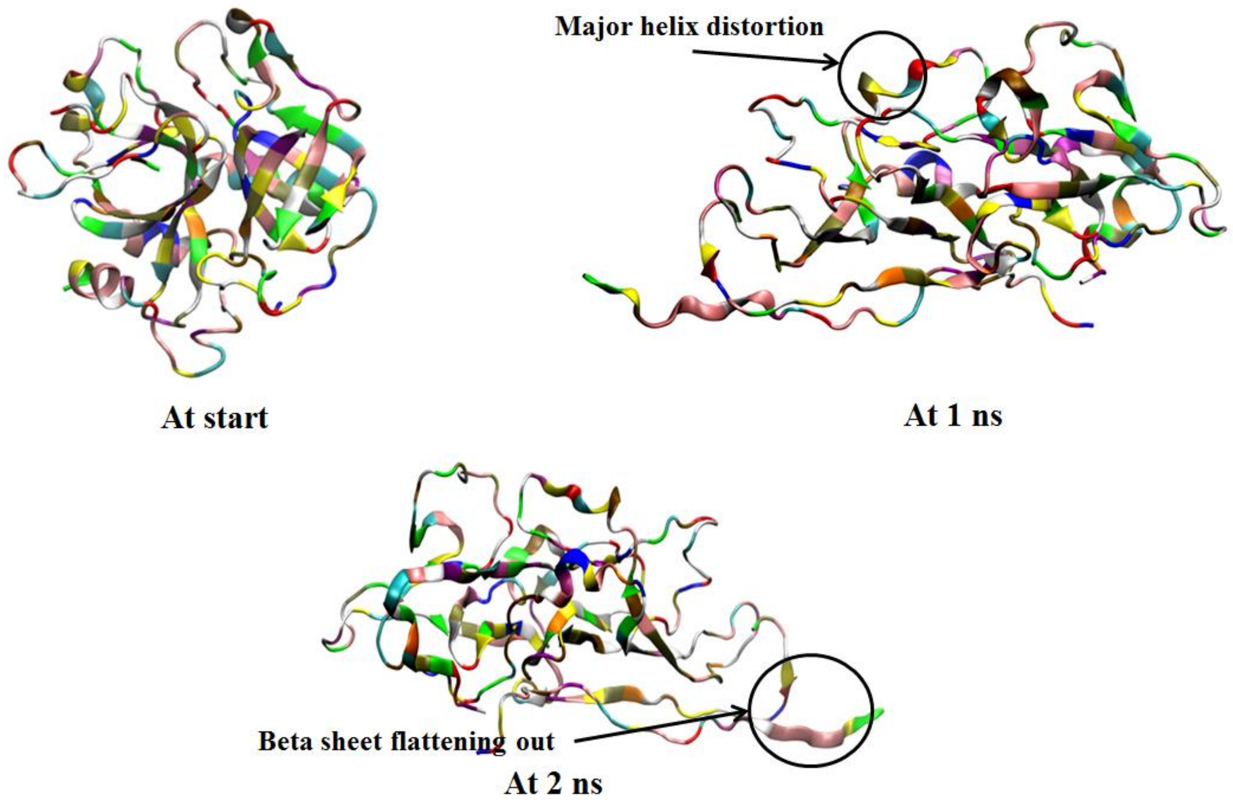

Figure S10

S10: The effect of (+) 3.0 Vnm^{-1} electric field on the structure of thrombin, as observed from the visualization of the corresponding MD simulation is shown. All images are rendered in VMD[3] . The protein structure deteriorates rapidly with increase in simulation time in presence of the strong electric field.

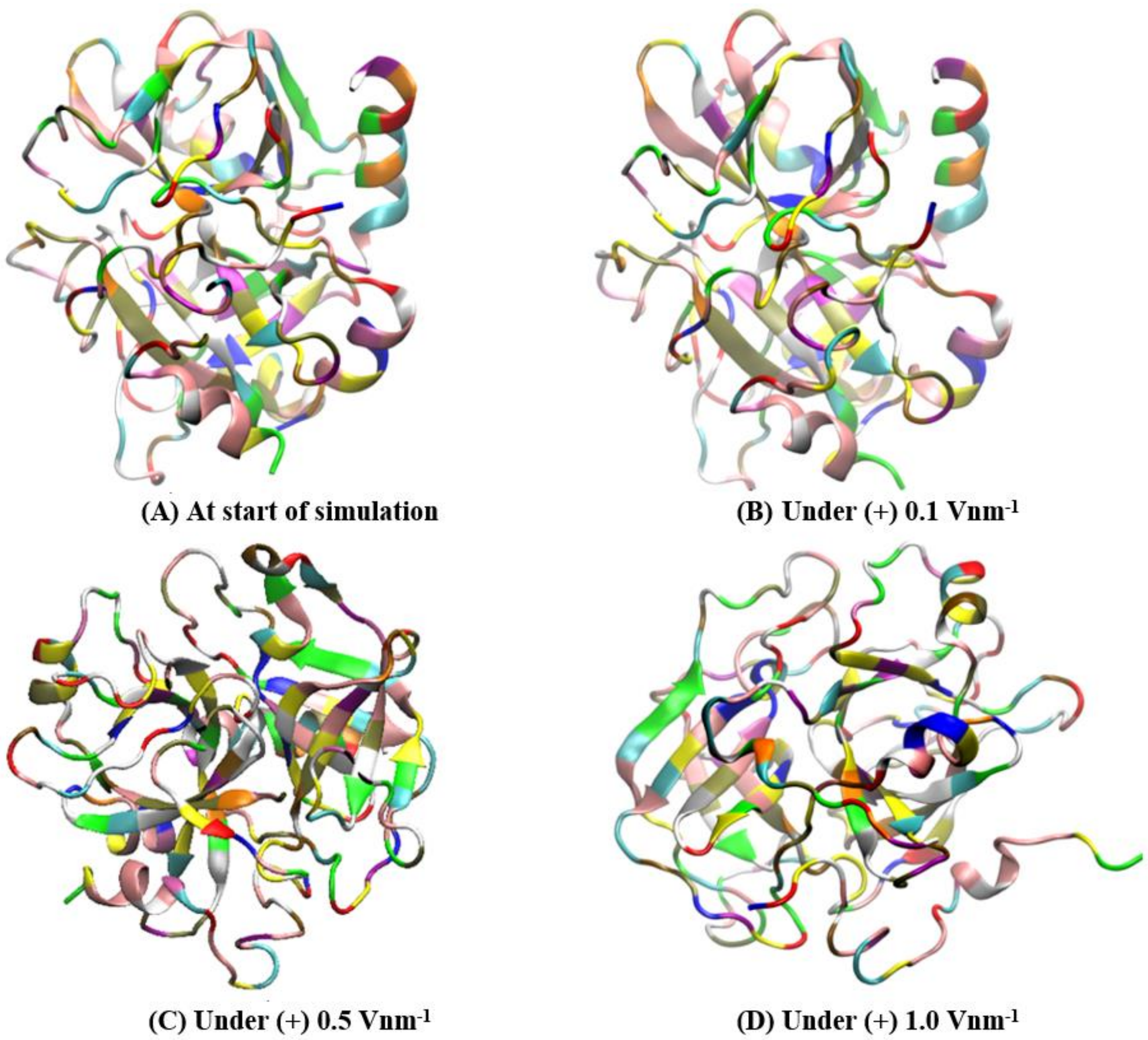

Figure S11

S11: The comparative images obtained from the visualization of the positive electric field simulation on the thrombin-aptamer complex is shown. The snapshots are produced from the end of the 5 ns simulation. The designation is as follows: A: 0 Vnm^{-1}, B: (+) 0.1 Vnm^{-1}, C: (+) 0.5 Vnm^{-1}, D: (+) 1.0 Vnm^{-1}. It is observed that at higher fields there is gradual unfolding of thrombin due to distortion in protein secondary structure. The alpha helices and the beta sheets are found to be distorted to a greater extent with increasing electric field magnitude. The unfolding of the protein results in higher RMSD values as observed in Fig 3 of the main article.

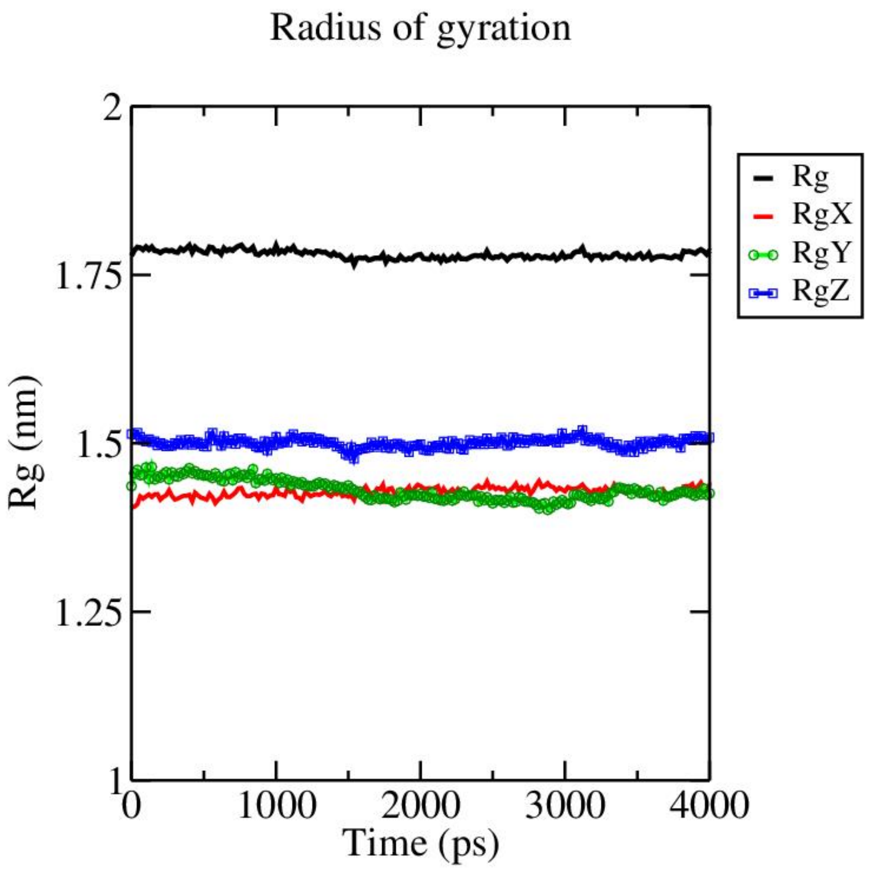

Figure S12

S12: The time evolution of radius of gyration (R_g) of the thrombin protein under **(+) 0.5 Vnm^{-1} electric field**, calculated for 4 ns of the 5 ns simulation, is shown. The thrombin exited the simulation box and hence the last 1 ns is excluded. The radius of gyration may be described as the mass weighted scalar length of each atom from the center-of-mass (COM). From the above figure it can be seen that the $R_g$remains fairly constant throughout the simulation time and this confirms that the globular protein structure did not experience unfolding and retained its shape under the effect of the electric field.

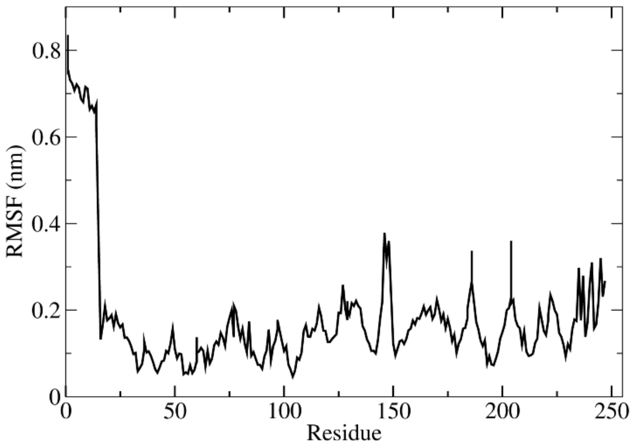

Figure S13

S13: The time evolution of RMSF (Root Mean Square Fluctuation) of thrombin residues under **(+) 0.5 Vnm^{-1} electric field** calculated for the last 1 ns of MD simulation time (while thrombin was inside the simulation box) is shown. RMSF is the time-average of RMSD (described in the main article) for each residue and shows the dynamic behavior of each residue. The plot shows that the thrombin residues, particularly those at the start of the backbone chain are fluctuating more indicating the effect of the stress induced by the electric field.

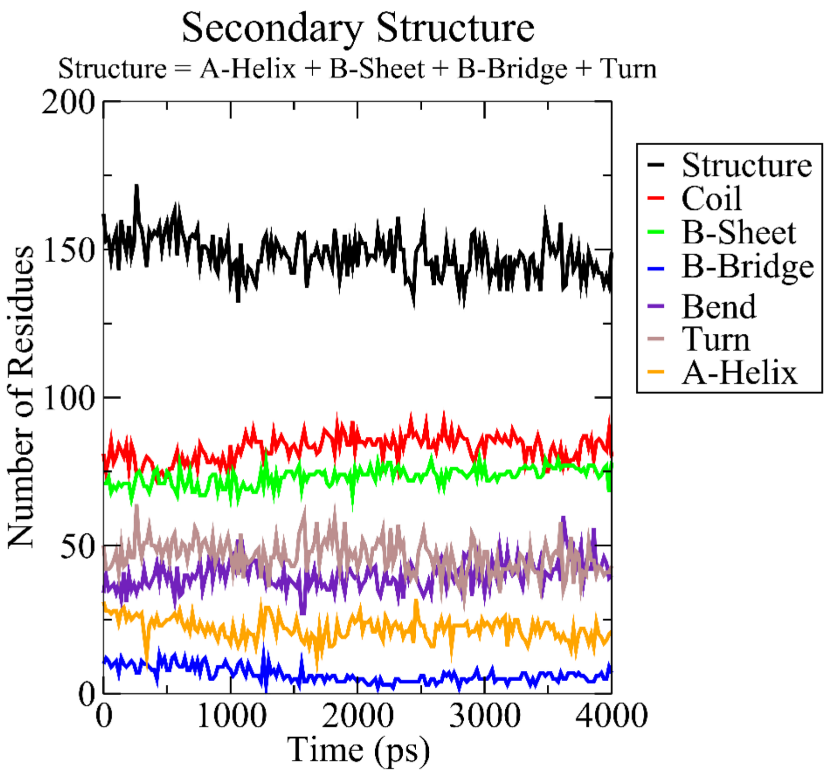

Figure S14

S14: The secondary structure evolution of thrombin, in presence of **(+) 0.5 Vnm^{-1}electric field** is shown for the different residues till 4 ns of the 5 ns simulation. The thrombin exited the simulation box and hence the last 1 ns is excluded. It is noticed that even if the RMSD (Figure 3 of main article) and RMSF (Figure S4) show higher values due to the application of a moderately high positive electric field, the secondary structure does not change much. The same can be observed visually from supplementary video S1. Secondary structure analysis is executed with the DSSP (Define Secondary Structure of Proteins) algorithm[12].

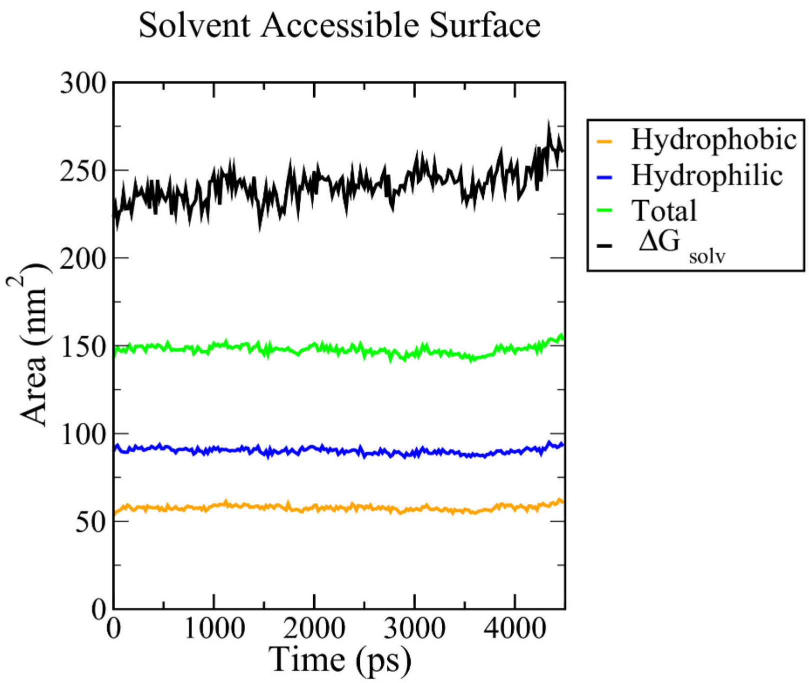

Figure S15

S15: The SASA (Solvent Accessible Surface Area) calculation is shown for thrombin under **(+) 0.5 Vnm^{-1} electric field**. The thrombin exited the simulation box, upon unbinding from the TBA, and hence the last 1 ns is excluded. It is observed that the magnitude of the total protein surface area is almost conserved indicating that there is no ingress of solvent due to protein unfolding and the globular structure is maintained. As expected the $\Delta G_{solvation}$ for thrombin with respect to the water (solvent) is also conserved throughout the simulation.

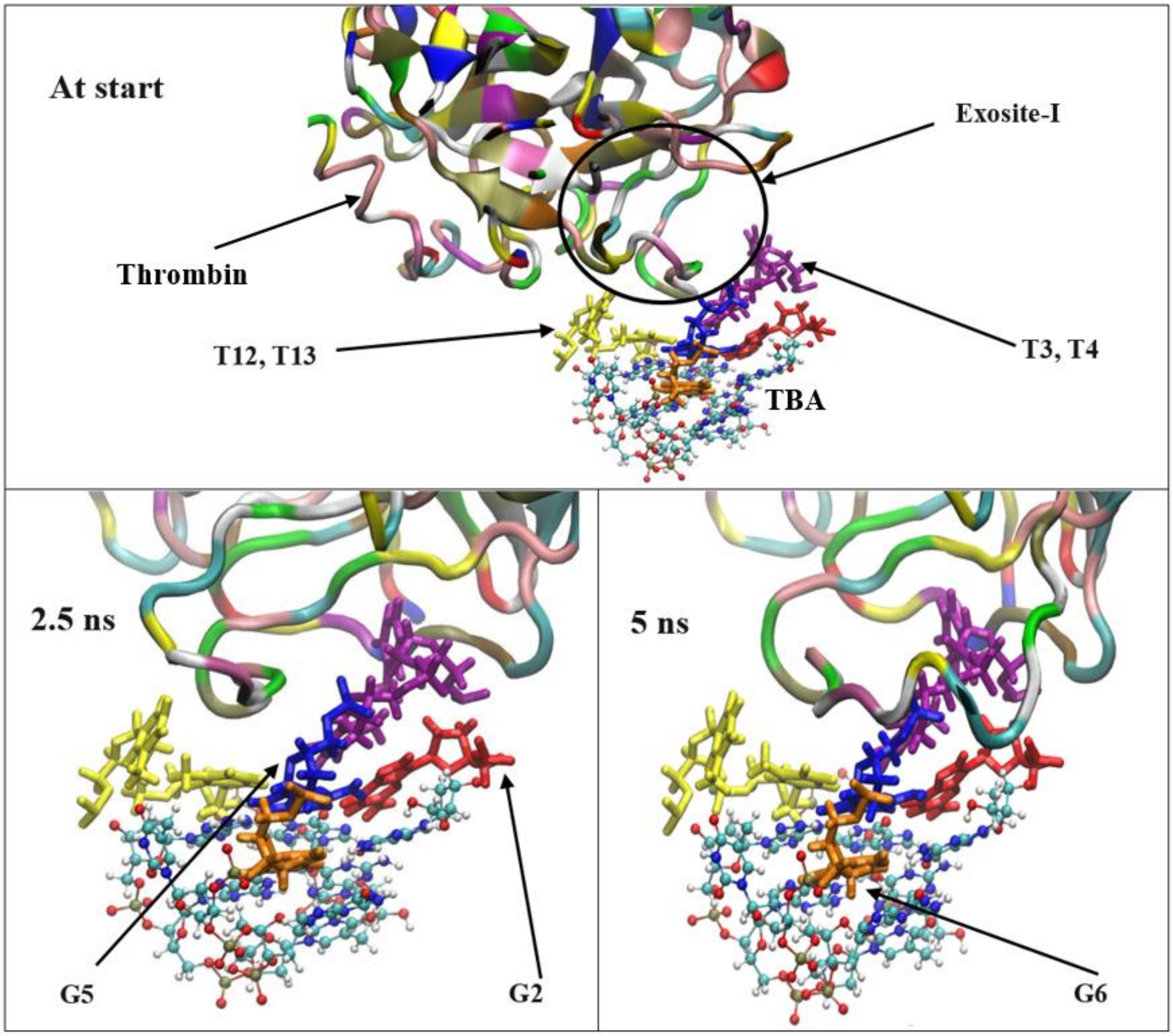

Figure S16

S16: The association between the exosite-I residues of thrombin and the TT loops as well as the G2,G5 and G6 bases is observed in the MD simulation with an electric field of (-) 1.0 Vnm^{-1}. Normally the TBA interacts with the exosite-I residues through its TT loops. In the simulation the TBA was position restrained and the protein was allowed to move freely. As the simulation progressed the protein reoriented itself such that many new interactions were promoted because of the close proximity of the G2, G5 and G6 bases to the reoriented protein. All images are rendered in VMD[3]. The thrombin is represented in the New Cartoon format whereas the T12, T13 (yellow), T3, T4 (purple), G2 (red), G5 (blue) and G6 (orange) bases are represented in the licorice format. All other TBA bases are represented in the CPK format. For clarity of visualization, water molecules and ions are removed.

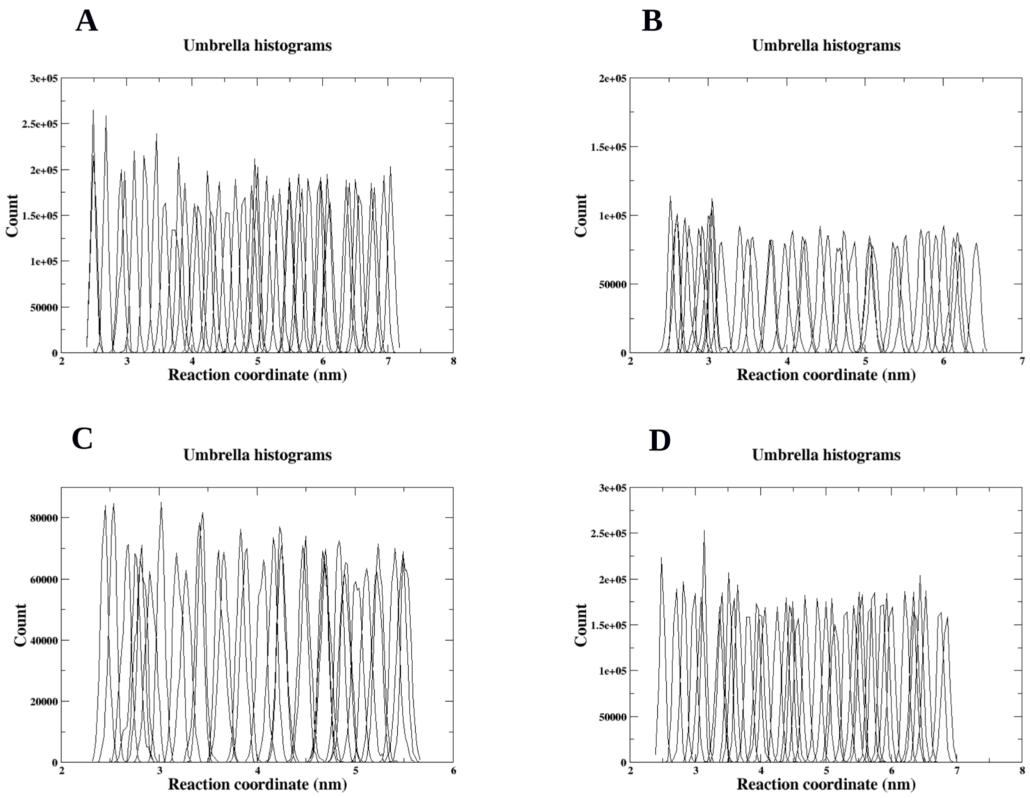

Figure S17 (a)

S17 (a): Umbrella sampling histograms for the different SMD simulations discussed in the article are given. The designation is as follows: A: 0 Vnm^{-1}, B: (+) 0.5 Vnm^{-1}, C: (+) 1.0 Vnm^{-1}, D: (-) 0.5 Vnm^{-1}.

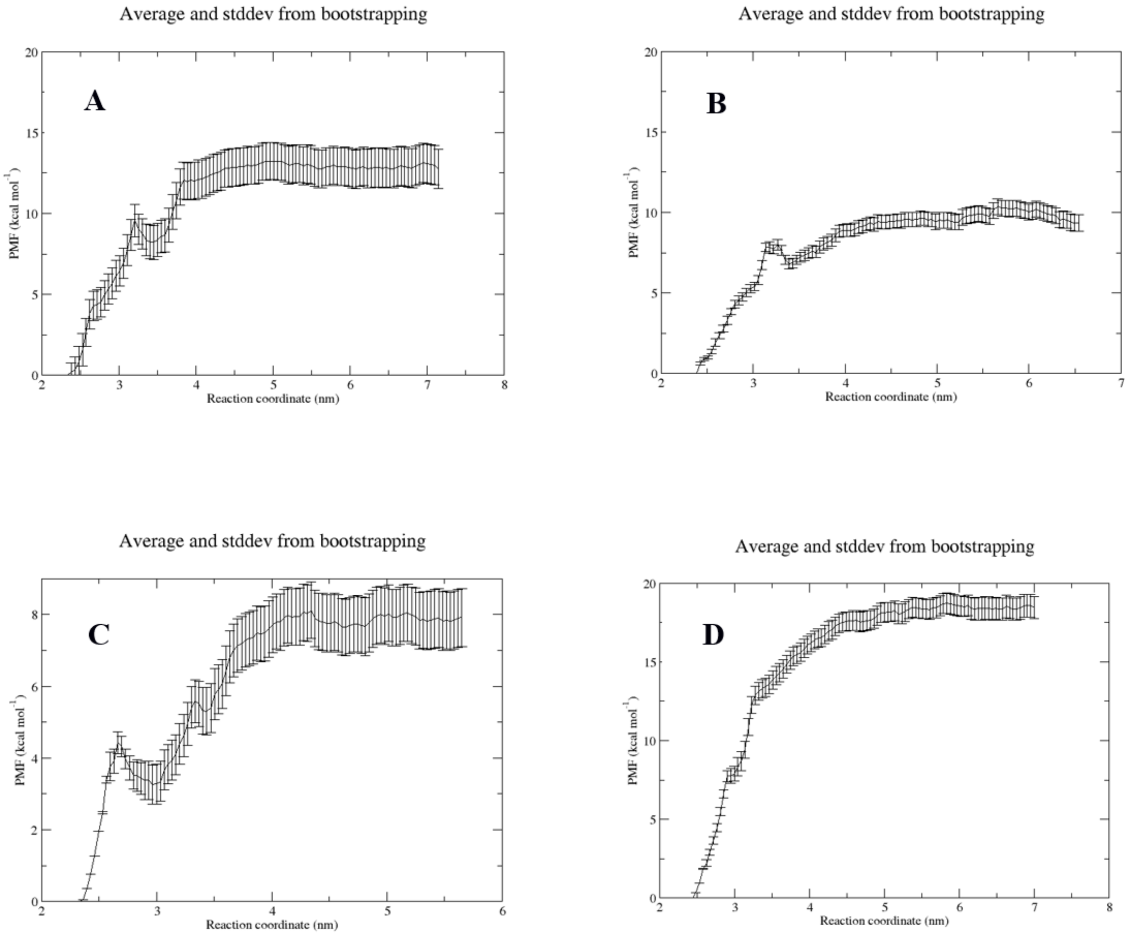

Figure S17 (b)

S17 (b): Umbrella sampling bootstrap results with 100 bins for the different SMD simulations discussed in the text are given. The designation is as follows: A: 0 Vnm^{-1}, B: (+) 0.5 Vnm^{-1}, C: (+) 1.0 Vnm^{-1}, D: (-) 0.5 Vnm^{-1}. The length of the error bars are expected to decrease in length with longer sampling times.

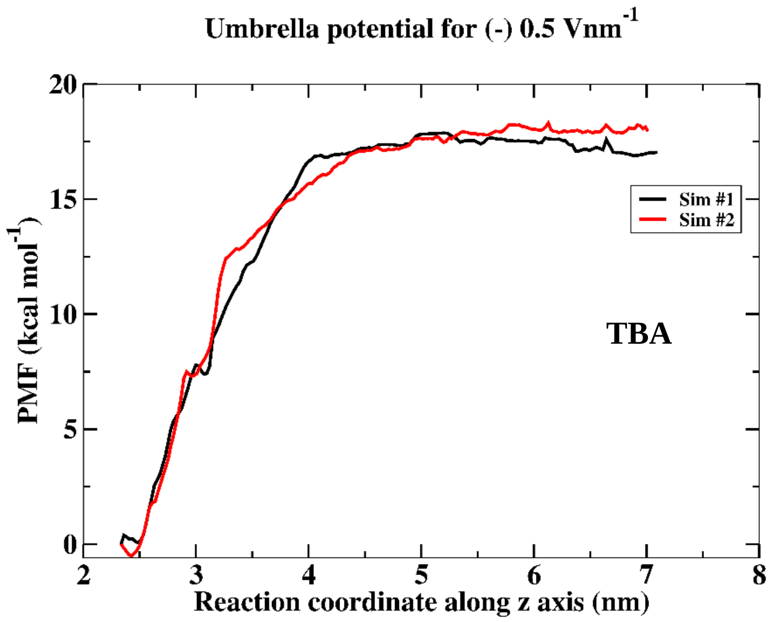

Figure S19

S19: Our results for two different US simulations with the same parameters conducted on two different SMD simulation trajectories with identical starting system are repeatable and reproducible.

Bibliography

1. Radi, A.-E., Acero Sánchez, J.L., Baldrich, E. & O'Sullivan, C.K. Reagentless, Reusable, Ultrasensitive Electrochemical Molecular Beacon Aptasensor. *Journal of the American Chemical Society* **128**, 117-124 (2006).
2. Ma, Xiao, "Electrostatic actuation based modulation of polar molecules and associated force interaction studies" (2013). *Graduate Theses and Dissertations.* Paper **13488**. (http://lib.dr.iastate.edu/etd/13488).3. Humphrey, W., Dalke, A. & Schulten, K. VMD: Visual molecular dynamics. *Journal of Molecular Graphics* **14**, 33-38 (1996).
4. Lemkul, J.A. & Bevan, D.R. Assessing the stability of protofibrils using molecular dynamics. *Journal of Physical Chemistry B* **114**, 1652-1660 (2010).
5. Pagano, B., Martino, L., Randazzo, A. & Giancola, C. Stability and binding properties of a modified thrombin binding aptamer. *Biophysical journal* **94**, 562-569 (2008).
6. Kim, E., Yang, C. & Pak, Y. Free-Energy Landscape of a Thrombin-Binding DNA Aptamer in Aqueous Environment. *Journal of Chemical Theory and Computation* **8**, 4845-4851 (2012).
7. Kästner, J. Umbrella sampling. *Wiley Interdisciplinary Reviews: Computational Molecular Science* **1**, 932-942 (2011).
8. Mills, M. & Andricioaei, I. An experimentally guided umbrella sampling protocol for biomolecules. *J Chem Phys* **129**, 114101 (2008).
9. Torrie, G.M. & Valleau, J.P. Nonphysical sampling distribution in Monte Carlo free energy estimation: umbrella sampling. *Journal of Computational Physics* **23**, 187-199 (1977).
10. Hub, J.S., Groot, B.L.D. & Spoel, D.V.D. g_wham-A Free Weighted Histogram Analysis Implementation Including Robust Error and Autocorrelation Estimates. *Journal of Chemical Theory and Computation* **6**, 3713-3720 (2010).
11. Maitz, M.F., Sperling, C. & Werner, C. Immobilization of the irreversible thrombin inhibitor D-Phe-Pro-Arg-chloromethylketone: A concept for hemocompatible surfaces? *Journal of Biomedical Materials Research Part A* **94A**, 905-912 (2010).
12. Kabsch, W. & Sander, C. Dictionary of protein secondary structure: Pattern recognition of hydrogen-bonded and geometrical features. *Biopolymers* **22**, 2577-2637 (1983).
